# Fair molecular feature selection unveils universally tumor lineage-informative methylation sites in colorectal cancer

**DOI:** 10.1093/bioinformatics/btaf237

**Published:** 2025-07-15

**Authors:** Xuan Cindy Li, Yuelin Liu, Alejandro A Schäffer, Stephen M Mount, S Cenk Sahinalp

**Affiliations:** Cancer Data Science Laboratory, Center for Cancer Research, National Cancer Institute, National Institutes of Health, Bethesda, MD, 20892, United States; Program in Computational Biology, Bioinformatics, and Genomics, University of Maryland, College Park, MD, 20740, United States; Cancer Data Science Laboratory, Center for Cancer Research, National Cancer Institute, National Institutes of Health, Bethesda, MD, 20892, United States; Department of Computer Science, University of Maryland, College Park, MD, 20740, United States; Cancer Data Science Laboratory, Center for Cancer Research, National Cancer Institute, National Institutes of Health, Bethesda, MD, 20892, United States; Program in Computational Biology, Bioinformatics, and Genomics, University of Maryland, College Park, MD, 20740, United States; Department of Cell Biology and Molecular Genetics, University of Maryland, College Park, MD, 20740, United States; Cancer Data Science Laboratory, Center for Cancer Research, National Cancer Institute, National Institutes of Health, Bethesda, MD, 20892, United States

## Abstract

**Motivation:**

In the era of precision medicine, performing comparative analysis over diverse patient populations is a fundamental step toward tailoring healthcare interventions. However, the aspect of fairly selecting molecular features across multiple patients is often overlooked.

**Results:**

To address this challenge, we introduce FALAFL (FAir muLti-sAmple Feature seLection), an algorithmic approach based on combinatorial optimization. FALAFL is designed to perform feature selection in sequencing data which ensures a balanced selection of features from *all* patient samples in a cohort. We have applied FALAFL to the problem of selecting lineage-informative CpG sites within a cohort of colorectal cancer patients subjected to low-coverage single-cell methylation sequencing. Our results demonstrate that FALAFL can rapidly and robustly determine the optimal set of CpG sites, which are each well covered by cells across the vast majority of the patients, while ensuring that in each patient, a large proportion of these sites have high read coverage. An analysis of the FALAFL-selected sites reveals that their tumor lineage-informativeness exhibits a strong correlation across a spectrum of diverse patient profiles. Furthermore, these universally lineage-informative sites are highly enriched in the inter-CpG island regions. We hope that FALAFL will aid in designing panels for diagnostic and prognostic purposes and help propel fair data science practices in the exploration of complex diseases.

**Availability and implementation:**

The source code is available at: https://github.com/algo-cancer/FALAFL.

## 1 Introduction

The pursuit of precision medicine has ushered in a promising era of tailored interventions and therapies, where the molecular characteristics of individual patients guide treatment strategies (Ashley 2016). Central to this pursuit is the task of molecular feature selection, a pivotal step toward discerning distinctions and commonalities among patient cohorts. However, a critical aspect often overlooked is the fair selection of molecular features across patient samples, such as when patients are subjected to single-cell sequencing with varying read depth. Achieving a balance between *all* patients in molecular feature selection is pivotal to prevent bias in an unsupervised manner.

Prior research recognized the importance of fair feature selection in biomedicine and other domains. In natural language processing, Rios and Bispo (2018) showed that fair feature selection in text classification tasks can prevent individual terms from dominating the classification process, improving downstream results. Khojasteh *et al.* (2023) applied a data-balancing technique derived from one-class support vector machines onto drug-target interaction data to enhance the prediction of drug-target interactions. These diverse applications point to a need for fair feature selection methods to maintain balance in the selection process. The balance, in turn, contributes to the robustness and fairness of the analysis and enhances the quality of the gained insights. There are several additional examples that introduced balance to feature selection in which the primary objective is to maximize accuracy, e.g. for binary classification or clustering. These studies show how to select features by trading off accuracy and fairness/balance, in either a supervised (Antelo-Collado *et al.* 2021) or unsupervised (Sharma *et al.* 2023) manner. However, none of the above-mentioned approaches are designed to address variability in data coverage, which is commonly observed in single-cell sequencing data; furthermore, they do not directly tackle the fair selection problem for molecular features.

**Figure 1.**
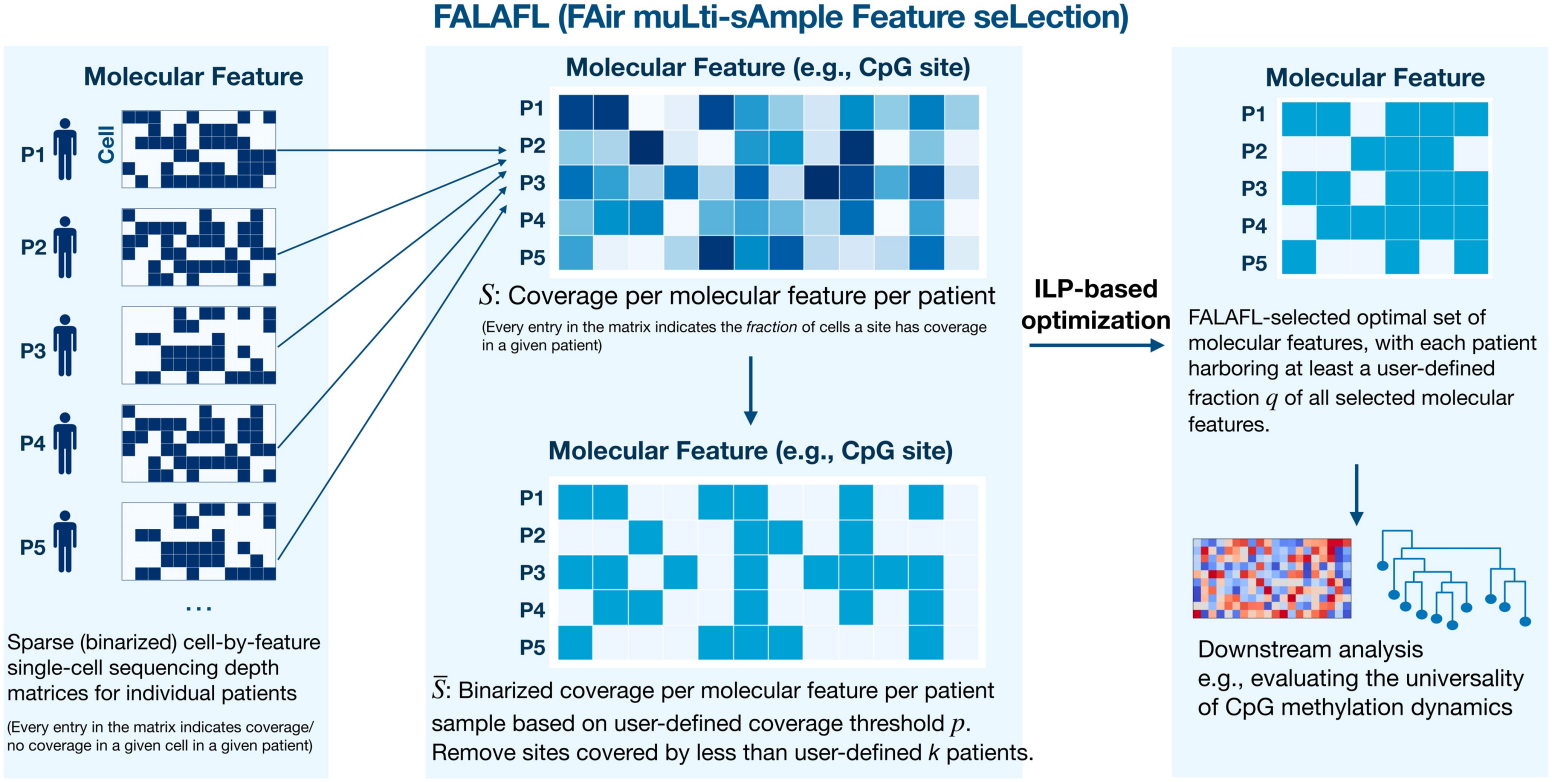
Graphical abstract of FALAFL. FALAFL takes the cohort molecular abundance profile and uses integer linear programming to output a set of molecular features that optimally represent the entire cohort. The FALAFL-selected features can then be used for downstream analysis, such as understanding the universality of lineage-informativeness of CpG sites in tumor progression.

Fair molecular feature selection across patient samples is related but orthogonal to the well-known “hitting set problem” (Karp 1972). The hitting set problem seeks to find the smallest possible set of elements H (referred to as the “hitting set”) from a given collection C of sets $C_{1},C_{2},\ldots$ such that at least one element $c_{i,j}$ from each set $C_{i}$ is included in H. In other words, this problem asks to identify a minimal set H that “hits” or intersects with each of the sets $C_{i}$ in the collection C.

The molecular feature selection problem has a similar setting but a very different objective. In this problem, each set $C_{i}$ for a given patient i is composed of elements $c_{i,j}$, each of which corresponds to a distinct molecular feature, such as a genetic marker, expression of a gene, or an epigenetic characteristic. To select a rich and balanced set of molecular features, one needs to come up with the *largest* subset of molecular features such that each feature is present in at least a user-defined number of patients, and each patient contributes nearly equally to the set of selected features. In real-world applications, the molecular feature selection problem is complicated by varying amounts of data (e.g. read depth) for each sample. For instance, in a colorectal cancer (CRC) single-cell dataset published by Bian *et al.* (2018), there is substantial variability in the number of cells collected from each patient and the coverage of CpG dinucleotide sites in each cell (see Supplementary Fig. S1). This observed variability within the cohort complicates the attempt to select CpG sites representing the entire cohort, thus obscuring the downstream efforts to understand epimutation dynamics across patients and identify driver methylation events. These real-world challenges call for a systematic approach to fairly select molecular features that encapsulate the entire patient cohort.

To overcome the said obstacles, in this paper, we introduce FALAFL (FAir muLti-sAmple Feature seLection, see Fig. 1), a novel fair molecular feature selection scheme grounded in integer linear programming (ILP). Designed to introduce algorithmic fairness in comparative patient profile analysis, the primary goal of FALAFL is to provide a fast and reliable solution for the balanced selection of molecular features across different patient samples to represent the *entire* patient cohort. When finding the optimal solution for feature selection, FALAFL not only achieves balance across all patients, but also ensures that each patient receives sufficient representation.

We have applied FALAFL to the problem of fair selection of CpG sites within the aforementioned CRC cohort (Bian *et al.* 2018), in which the patients are subjected to low-coverage single-cell methylation sequencing. We demonstrate that FALAFL rapidly and robustly identifies a maximal set of CpG sites that are well-represented across a majority of patients, while ensuring that each patient contributes a proportionate number of sites to the final selection, enabling fair comparative analysis of the selected sites. We then performed such a comparative downstream analysis of the FALAFL-selected sites, which revealed that lineage-informativeness among the CpG sites, i.e. the property that the methylation status of a CpG site is stably inherited in tumor progression, is universal. These findings unveil the universal non-stochastic nature of methylation changes in the course of colorectal cancer progression.

## 2 Materials and methods

The input to FALAFL is genomic, epigenomic, or transcriptomic sequencing data from a set of patient samples. For the remainder of the paper, we will focus on single-cell methylation sequencing data, but FALAFL is also applicable to other types of sequencing data such as single nucleotide polymorphisms (Phuong *et al.* 2005, Nassir *et al.* 2009). In single-cell methylation sequencing, the average read depth across any given cell’s genome is very low (0.1× or lower) and highly variable across the CpG sites, cells, and samples. Nevertheless, because methylation statuses of many CpG sites are altered during tumor evolution, there have been attempts to reconstruct a tumor’s progression history from single-cell methylation sequencing data (Bian *et al.* 2018, Gaiti *et al.* 2019).

Only a subset of CpG sites in a tumor are “lineage-informative,” meaning that such sites have some variation in methylation status between cells, and the differences can be associated with distinct lineages in a tumor phylogeny. It is possible to identify lineage-informative sites in a tumor sample, while simultaneously inferring the tumor phylogeny, by an iterative approach named Sgootr (Liu *et al.* 2023). Sgootr has been applied to the single-cell methylation sequencing dataset from several colorectal cancer patients (Bian *et al.* 2018) to demonstrate that the numbers of distinct metastatic seeding events in these tumors are fewer than those reported in the original study. However, because Sgootr only uses CpG sites that are well covered across the cells of a given tumor sample, the set of CpG sites Sgootr identified as lineage-informative in different samples vary considerably. This makes it difficult to assess whether lineage-informativeness of CpG sites is shared across all or a subset/subtype of colorectal cancer or is sample-specific. Such an assessment is important since recurrent lineage-informativeness could imply a functional association between lineage-informative CpG sites and colorectal cancer evolution.

FALAFL takes as input *the read depth information* (note that read depth is different from methylation status) for each CpG site, in each cell, from each tumor sample, and outputs a set of CpG sites, each with sufficient read depth across the cells of nearly all (i.e. except a small number of) patients. Importantly, FALAFL identifies CpG sites that are balanced across tumor samples, in the sense that, the proportion of these CpG sites with sufficient read depth across a large proportion of cells in each tumor sample will be higher than a user-defined threshold. On the CpG sites selected by FALAFL, one can evaluate lineage-informativeness in each tumor sample and assess whether this property is shared between samples.

### 2.1 FALAFL: fair selection of molecular features for multi-patient comparative analysis

The input on which we test FALAFL is multi-patient integrated CpG site read coverage data represented as a patient-by-site matrix $S_{n\times m}$, where n is the number of patients, m the number of CpG sites, and $s_{i,j}$ the fraction of cells in patient (i.e. tumor sample) i in which CpG site j has “sufficient” read depth (e.g. two reads or more) as defined by the user.

In the first preprocessing step, we ensure that for each patient i, we have $s_{i,j}\geq \delta$ (for some user-defined δ) for each site j; if not, site j is not considered any further for that patient. This preprocessing step reduces the size of the input matrix and is optional. The intuition is that we want the sites eligible for subsequent analysis steps to have more evidence from more reads.

In the second preprocessing step, we binarize S to obtain $\bar{S}$, where ${\bar{s}}_{i,j}$ indicates whether site j has sufficient read depth in at least a fraction of p cells in patient i. The second step conceptually simplifies the problem by changing a set of integer variables to 0/1 variables.


(1) $${\bar{s}}_{i,j}=\{\begin{array}{cc} 1 & \text{if}\;s_{i,j}\geq p\;\text{in}\;\text{patient}\;i\\ 0 & \text{otherwise} \end{array}$$

In the third preprocessing step, we eliminate all sites j where the total number of patients i with $s_{i,j}=1$ is <k, for some user-defined threshold k. The intuition is that we reduce the size of the problem by eliminating from consideration sites that have too little evidence of the methylation status for too many patients. We achieve this by removing all sites j for which ${\bar{s}}_{j}=0$:


(2) $${\bar{s}}_{j}=\{\begin{array}{cc} 1 & \text{if}\;\sum_{i=1}^{n}{\bar{s}}_{i,j}\geq k\\ 0 & \text{otherwise} \end{array}$$

After preprocessing, we define our combinatorial optimization formulation, namely *the FAir muLti-sAmple Feature seLection* (FALAFL) problem, on the preprocessed binary input matrix ${\bar{S}}_{n,m^{\prime }}$ (where $m^{\prime }$ denotes the number of sites not eliminated in the preprocessing steps) as follows. Choose the largest subset of sites $s_{j}$, such that, for each patient i, the proportion of ${\bar{s}}_{i,j}=1$ among the chosen sites is at least q. Below, we first give an ILP (integer linear programming) formulation to solve the FALAFL problem after preprocessing and then demonstrate that the problem is NP-hard.

#### 2.1.1 An ILP formulation for the FALAFL problem

Let r be a binary vector where $r_{j}=1$ indicates that site j is chosen by the formulation. Then our formulation chooses sites with the objective to

maximize:


(3) $$\sum_{j=1}^{m^{\prime }}r_{j}\sum_{i=1}^{n}{\bar{s}}_{i,j}$$

subject to the constraint that:


(4) $$\forall i\in \{1\ldots n\}:\sum_{j=1}^{m^{\prime }}r_{j}\cdot {\bar{s}}_{i,j}\geq q\cdot \sum_{j=1}^{m^{\prime }}r_{j}$$

The above formulation of FALAFL is implemented using the Gurobi ILP solver (Gurobi Optimization, LLC 2022). The implementation of FALAFL is available on Github (https://github.com/algo-cancer/FALAFL).

#### 2.1.2 Proof of NP-hardness of the FALAFL problem

We now prove that the FALAFL problem is NP-hard. For that, we give a polynomial (time/space) reduction from the *Exact 3-Cover* Problem (abbreviated X3C), which is NP-hard (Garey and Johnson 1979).

Lemma 1. The FALAFL problem is NP-hard.

Proof.

The X3C problem is defined as follows. Given a collection S of three-element subsets of a set X whose size is a multiple of 3, an exact cover is a subcollection $S^{*}\subset S$ such that each element in X is contained in (i.e. “covered by”) exactly one subset in $S^{*}$. The X3C problem asks whether an exact cover $S^{*}\subset S$ exists for X. If the size of X is 3t, then the number of sets in the exact cover $S^{*}$ must be t.

We give a reduction from X3C to the decision version of the FALAFL problem: given a binary matrix Q, is there a set of exactly k columns such that each row has at least ℓ 1s in these columns (an algorithm that can solve the decision version of the FALAFL problem in polynomial time can also solve the optimization version of the FALAFL problem by solving the decision version for different values of k).

Suppose that we are given an instance of X3C where $X=\{x_{1},\ldots ,x_{\alpha }\}$, so that |X|=α and $S=\{S_{1},\ldots ,S_{\beta }\}$, so that |S|=β. To reduce this problem instance to an instance of the decision version of the FALAFL problem, create an α×β sized binary matrix Q as follows. $Q_{i,j}=1$ if and only if $x_{i}\in S_{j}$. Now assign k=α/3 and ℓ=1.

We now prove that the answer to the given instance of the X3C problem is “true” if and only if the answer to the instance for the decision version of the FALAFL problem is “true.” Suppose that the answer to the given instance X3C problem is “true”; this means that there is a collection $S^{*}$ with $|S^{*}|=\alpha /3$ where each element $x_{i}\in X$ is present in only one set $S_{j}\in S^{*}$. Thus, there are k=α/3 columns of Q, each corresponding to a distinct set $S_{j}\in S^{*}$, so that each row i has value 1 in exactly one of these columns, which implies that these columns form a solution to the instance of the FALAFL problem. Conversely, suppose that the answer to the decision version of the FALAFL problem is “true”; this implies that there are k columns in Q, among which each row i has value 1 in at least one column. Since k=α/3, this means that the chosen columns “cover” 3×α/3=α rows, indicating that the sets they correspond to “cover” each element of X exactly once. □

### 2.2 Evaluating lineage-informativeness of FALAFL-selected CpG sites from colorectal cancer patients by using tumor phylogenies

A single-cell methylation sequencing (more specifically, single-cell bisulfite sequencing, or sc-BSseq) dataset has been made available by Bian *et al.* (2018) for nine metastatic colorectal cancer patients. A description of the number of cells, number of input CpG sites, and lesion information for each patient is in Supplementary Table S1. The distribution of the number of CpG sites sequenced per cell in each patient can be found in Supplementary Fig. S1. As will be described in detail in Section 3.2, we apply FALAFL to the single-cell methylation sequencing data from these nine patients to identify ∼196K CpG sites that are well covered across the cells of a large fraction of patients, while ensuring that a large fraction of these sites are well covered in each of the patients. We also apply FALAFL to identify ∼1.35M CpG sites from four patients with a primary tumor in the left colon; these patients have better cell coverage and read depth than the others. On these CpG sites, we evaluate the lineage-informativeness in each patient as described below.

For each patient, on each CpG site i, consider the reads in cell j that cover i; let N(i,j) be the number of those reads where i is methylated and M(i,j) be the number of those reads where i is unmethylated. Given these values, respectively forming “read count matrices” N and M, we apply Sgootr (Liu *et al.* 2023), to infer a tumor phylogeny for that patient, where each leaf represents a single cell. Sgootr additionally identifies “lineage-informative sites” for that patient: these are the CpG sites whose methylation status emulates the infinite sites assumption, i.e. these are the sites whose methylation status is altered only once in the tumor phylogeny. Specifically, Sgootr identifies these lineage-informative sites by calculating for each internal node of the tumor phylogeny, the Jensen-Shannon (JS) distance (Wong and You 1985, Lin 1991) between the site’s methylation level distribution (i) across the cells in the clade rooted at that node, and (ii) the remainder of the tree. A high JS distance for a site in a given node indicates that the methylation status of the site was possibly altered in that node during tumor evolution and nowhere else in the tree. Sgootr then returns the maximum JS distance across the internal nodes as the lineage-informativeness of the site in that patient. In the remainder of this section, we focus only on the lineage-informativeness of those sites i which have been selected by FALAFL.

### 2.3 Evaluating lineage-informative universality via deviation from perfection correlation

The lineage-informativeness of a site j in each patient is measured by the JS distance and ranges from 0 to 1. Across all n patients, the lineage-informativeness of site j can be represented as a vector $v_{j}$ of size n:


$$v_{j}=(v_{1,j},v_{2,j},\ldots ,v_{n,j})$$

We simplify the notation to $v_{j}$ as $v=(v_{1},v_{2},\ldots ,v_{n})$.

The universality of lineage-informativeness of CpG site j across all n patients can thus be evaluated as the $L_{2}$ distance between the point $V\in R^{n}$ with coordinates $\left(v_{1},v_{2},\ldots ,v_{n}\right)$ (which corresponds to the vector v), and the line ℓ of perfect equality, between the points $\ell_{0}=(0,0,\ldots ,0)$ and $\ell_{1}=(1,1,\ldots ,1)$, where $\ell_{0},\ell_{1}\in R^{n}$. Note that if the CpG site is truly universal, i.e. has identical methylation persistence across all patients, then v would be on the line of perfect equality.

Let n be the unit vector parallel to ℓ, i.e. $n=\frac{\ell_{1}}{\left||\ell_{1}|\right|}$, then the $L_{2}$ distance between V and ℓ can be computed as follows:


(5) $$\begin{array}{l} \mathrm{distance}(\ell ,V)=||v-(v\cdot n)n||\\ =||(v_{1},v_{2},\ldots ,v_{n})-\frac{1}{\sqrt{n}}(v_{1}+v_{2}+\cdots +v_{n})(\frac{1}{\sqrt{n}},\frac{1}{\sqrt{n}},\ldots ,\frac{1}{\sqrt{n}})||\\ =||(v_{1},v_{2},\ldots ,v_{n})-\frac{1}{n}(\sum_{i=1}^{n}v_{i},\sum_{i=1}^{n}v_{i},\ldots ,\sum_{i=1}^{n}v_{i})||\\ =||(v_{1}-\frac{1}{n}\sum_{i=1}^{n}v_{i},v_{2}-\frac{1}{n}\sum_{i=1}^{n}v_{i},\ldots ,v_{n}-\frac{1}{n}\sum_{i=1}^{n}v_{i})||\\ =\sqrt{{\left(v_{1}-\frac{1}{n}\sum_{i=1}^{n}v_{i}\right)}^{2}+{\left(v_{2}-\frac{1}{n}\sum_{i=1}^{n}v_{i}\right)}^{2}+\cdots +{\left(v_{n}-\frac{1}{n}\sum_{i=1}^{n}v_{i}\right)}^{2}}\\ =\sqrt{\sum_{i=1}^{n}{\left(v_{i}-\frac{1}{n}\sum_{i=1}^{n}v_{i}\right)}^{2}}\\ =\sqrt{\sum_{i=1}^{n}{\left(v_{i}-\hat{v_{i}}\right)}^{2}} \end{array}$$

Thus, distance(ℓ,V) is equivalent to the mean square difference between vector v treated as a point and the nearest point on the line of perfect identity. We normalize the resulting distance using the maximum distance possible in the n-dimensional Euclidean unit hypercube confined by $0\leq v_{i}\leq 1$ in the ith dimension for all n dimensions. The normalization yields a measure that is independent of the number of patients, which enables us to compare the universality of lineage-informativeness across all nine patients with that among those patients within each specific tumor subtype.

The mean square difference is maximized when the coordinates of $v_{i}$ are as dispersed as possible, i.e. half of $v_{i}$ are 0 and the other half are 1. If n is an even number, the maximum mean square difference of vector v is $\frac{\sqrt{n}}{2}$. If n is an odd number, the maximum mean square difference of vector is then $\sqrt{(\frac{n-1}{2}){\left(\frac{n+1}{2n}\right)}^{2}+(\frac{n+1}{2}){\left(\frac{n-1}{2n}\right)}^{2}}=\sqrt{\frac{\left(n+1)(n-1\right)}{4n}}$. Therefore, the maximum distance(ℓ,V) is the following:


(6) $$\mathrm{distance}{\left(\ell ,V\right)}_{\max}=\{\begin{array}{cc} \frac{\sqrt{n}}{2} & \text{if}\;n\in 2Z\\ \sqrt{\frac{\left(n+1)(n-1\right)}{4n}} & \text{otherwise} \end{array}$$

The normalized distance, which we call *deviation from perfect correlation*, is the following:


(7) $$\tilde{\mathrm{distance}(\ell ,V)}=\frac{\mathrm{distance}(\ell ,V)}{\mathrm{distance}{\left(\ell ,V\right)}_{\max}}$$

### 2.4 Defining universally lineage-informative and uninformative CpG sites

The lineage-informativeness of a CpG site characterizes how stably inherited its methylation change is in a patient, and the universality of lineage-informativeness measures how conserved its methylation change dynamics are across different patients. Lineage-informativeness is determined by the mean JS distance of the site across patients. The universality of a site’s behavior across patients is determined by its deviation from perfect correlation, as described in Section 2.3. Universally lineage-informative sites have a large mean JS distance across all patients but exhibit only a small deviation from perfect correlation. Conversely, universally uninformative sites display both a small mean JS distance across all patients and a small deviation from perfect correlation.

Specifically, we deem a CpG site universally lineage-informative, if its mean JS distance exceeds the mean value of all sites’ mean JS distances across patients, and its deviation from perfect correlation is smaller than the mean perfect correlation of sites. On the contrary, if a site’s mean JS distance is smaller than the mean value of all sites’ mean JS distances across patients, and its deviation from perfect correlation is smaller than the mean perfect correlation of all sites, then it is classified as universally uninformative.

### 2.5 Benchmarking FALAFL with naive feature selection approaches

We conduct three different types of naive feature selection approaches to benchmark against FALAFL. (i) We first benchmark FALAFL against the naive measure of selecting well-covered sites in a high fraction of patients. Specifically, we select sites with good coverage in at least half of the patients in the cohort of interest. In the left CRC cohort, we select sites well covered in at least two patients, and in the entire CRC cohort, we select sites well covered in at least five patients. (ii) The second naive approach we use is greedy pairwise selection, which seeks to select CpG sites to represent a given patient pair. For each patient pair, sites covered by at least one read in 50% or more of the cells of both patients are selected. (iii) The last naive approach used for benchmarking is random selection, whereby the same number of sites as FALAFL output for the 4-patient left CRC patient subcohort (i.e. 1 346 130 sites) are randomly chosen from the unpreprocessed 27 227 230 sites. The random selection is repeated five times. Both the greedy pairwise selection and random selection are conducted only specifically on the subcohort of four patients with the primary tumor in the left colon, primarily because these patients have higher numbers of cells and better read depth coverage. Additionally, we would like to perform the benchmarking analysis within a homogeneous patient cohort to avoid potential perturbations due to high levels of patient diversity as observed in the entire patient cohort.

## 3 Results

We apply FALAFL to the scBS-seq dataset (Bian *et al.* 2018) generated by Bian *et al.* on nine metastatic colorectal cancer patients. While the Bian *et al.* cohort consist of 12 patients in total, we choose to exclude patients CRC03 and CRC06 for not having scBS-seq data and CRC09 for not having metastasis cells with scBS-seq data. We acknowledge that excluding patients such as CRC09 due to phenotypic characteristics may introduce bias in the results. In this cohort, patients CRC01, CRC10, CRC11, and CRC13 have primary tumor sites in the left colon, CRC02, CRC04, and CRC15 in the right colon, and CRC12 and CRC14 in the rectum. Here, we present results generated from applying FALAFL to the entire CRC cohort as well as the subcohort of four patients whose primary tumor is in the left colon (i.e. left colon CRC subcohort). We also include the results obtained from benchmarking FALAFL against the naive selection approaches described in Section 2.5.

To demonstrate its generalizability, we also apply FALAFL to a scRRBS (Reduced Representation Bisulfite Sequencing) dataset by Chaligne *et al.* on 11 brain cancer (glioma) patients. See Supplementary Section S3 for results on this dataset.

### 3.1 FALAFL scales on different numbers of patients

**Figure 2.**
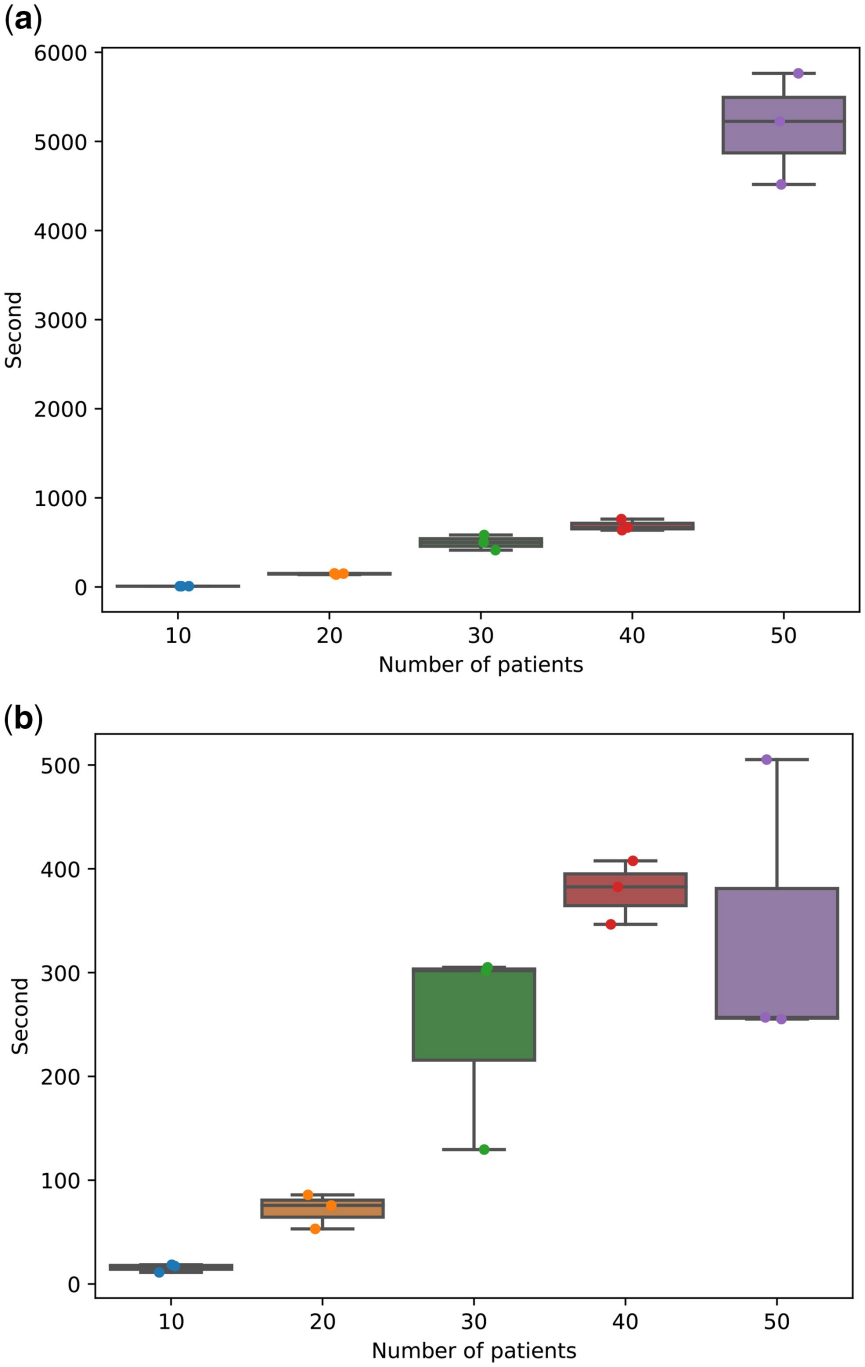
FALAFL runtime on simulated data across different numbers of patients. The boxplots show time spent to obtain optimal solutions in (a) randomly generated binary matrices and (b) randomly perturbed real patient data. The longer runtime for simulated data is possibly spent on the branch-and-bound strategy utilized by Gurobi in exploring a solution space that consists of many near optimal solutions.

To evaluate the scalability of FALAFL on different numbers of patients in the input, we conduct experiments on runtime performance using both synthetic and perturbed real patient data. We first assess the runtime of FALAFL on randomly generated binary matrices with increasing numbers of patients. Random binary matrices were generated for 10, 20, 30, 40, and 50 patients, each with 1 million sites. The probability of a site being 1 was set to 0.66, reflecting real data characteristics. For each patient count, three random instances were created. The time spent (in seconds) to obtain the optimal solution is summarized in Supplementary Table S2 and Fig. 2a. We then evaluate the runtime of FALAFL using perturbed real patient data. After filtering with δ=0.1, p=0.5 and k=4, the real patient data binary matrix for the entire CRC cohort is sized at 9 × 1 175 877. We randomly select from the nine patients to form binary matrices with 10, 20, 30, 40, or 50 patients. We then perturb the entries in the binary matrix by flipping 0 to 1 or 1 to 0 with a probability of 0.1. Again, for each patient count, we create three random instances. The ILP-based feature selection parameter is set to q=0.75. The time spent (in seconds) to obtain the optimal solution is summarized in Supplementary Table S3 and Fig. 2b. These results indicate that FALAFL is capable of scaling to input with larger numbers of patients, with runtime reasonably increasing as the number of patients grows. Additionally, we demonstrate that different choices of q have a very small impact on running time, as can be seen in Supplementary Table S4. All experiments are conducted on a single CPU.

### 3.2 FALAFL performs fast and robust molecular feature selection on real sequencing data

We prepare the patient data from the left colon CRC subcohort as well as the entire patient cohort for preprocessing into the input matrix tailored for FALAFL. For both cohorts, we choose a value of δ=0.1, indicating that a CpG site needs to be covered by at least one read in at least 10% of the cells in each of the patients. This results in 27 227 230 sites for the left colon CRC subcohort and 18 991 006 sites for the entire CRC cohort, respectively. We empirically chose 0.5 for p and the floor of half of the cohort size for k, as these are neutral starting points when no additional information is available. We chose 0.75 for q, as the input data matrix is 66% covered, and therefore, 0.75 is a reasonable optimization goal.

Specific to the left colon CRC subcohort of four patients, we configure the input parameters for preprocessing as p=0.5 and k=2, indicating that a CpG site can be considered by FALAFL only if it has at least one read covering it in more than 50% of the cells in two or more patients. This results in the generation of two preprocessed input matrices of the same dimensions, denoted as S and $\bar{S}$, as described in Section 2.1. For the left colon CRC subcohort, the preprocessed input matrices are sized at 4×2 133 129. We choose q=0.75 as the input parameter for the ILP constraint as defined in Equation (4), which indicates that in the output matrix, each patient needs to have sufficient coverage in at least 75% of the FALAFL-selected sites.

Specific to the entire CRC cohort of nine patients, we use p=0.5 and k=4 as the input parameters for preprocessing. As the nine patients have different primary tumor sites and sequencing coverage, we further add a constraint that each CpG site must have coverage in at least two left colon CRC patients, one right colon CRC patient, and one rectum CRC patient. This yields two preprocessed input matrices S and $\bar{S}$, both sized at 9×1 175 877. The choice for the input parameter for the ILP constraint is q=0.75.

Despite the large dimensionality of the input data, FALAFL manages to deliver outputs almost instantly. On a single CPU, FALAFL completes feature selection for both the left colon CRC subcohort and the entire CRC patient cohort in <60 s, underscoring the computational efficiency of our approach. For the left colon CRC subcohort, FALAFL yields an output consisting of 1 346 130 selected sites for the four patients; for the entire CRC patient cohort, FALAFL select 195 809 sites for the nine patients. For each patient cohort, the application of FALAFL on the respective input matrix is repeated three times, and the runtime is shown in Supplementary Fig. S2a.

**Figure 3.**
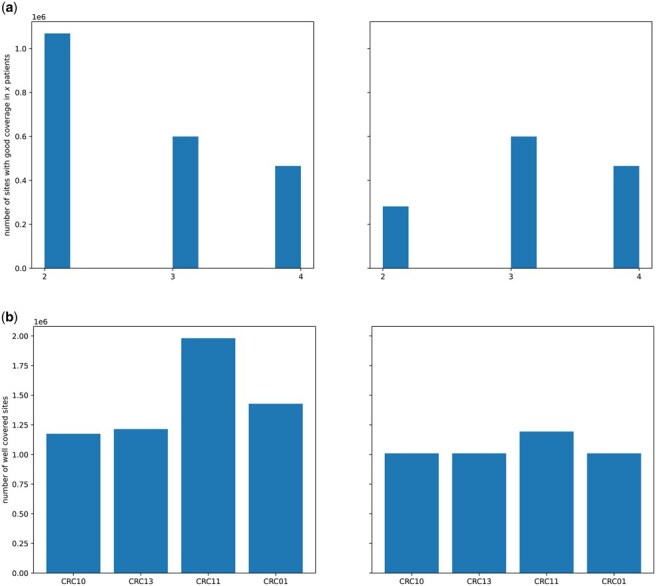
FALAFL reduces bias toward individual patients in the left CRC cohort. (a) Bar plot showing the number of sites with good coverage in different number of patients. (b) Bar plot showing the number of sites with good coverage in each patient sample. The left panels show the results from naively selected sites with good coverage in two or more patients. The right panels show the results from FALAFL-chosen sites.

To evaluate the robustness of our output, we perform three instances of data shuffling on the input matrix for the 4-patient left colon CRC subcohort. More specifically, we randomly shuffle the order of the columns of the input matrix to assess whether FALAFL favors certain columns over others when producing outputs. We compare three experiments of data shuffling in pairs: for each pair of experiments, we evaluate the similarity of the FALAFL-outputted sites using the Jaccard index. As indicated in Supplementary Fig. S2b, the results demonstrate a high level of consistency—for all three pairs of the data shuffling experiments, the outputs of FALAFL overlap highly, with the Jaccard index ranging from 0.9999 to 1.0. The high degree of agreement among the outputs further attests to the reliability and stability of our selection process, signifying the consistency and reproducibility of FALAFL’s results.

### 3.3 FALAFL performs balanced feature selection compared to naive approaches

We benchmarked FALAFL-selected sites against sites selected using the naive approach of selecting sites present in a high fraction of patients as described in Section 2.5. As can be seen in Fig. 3b, in the left CRC cohort, the naively chosen sites are heavily biased toward patient CRC11—CRC11 has notably more sites with good coverage than the other patients, whereas among FALAFL-chosen sites, the bias is reduced. The same observations can be made in the entire CRC cohort, as demonstrated in Supplementary Fig. S3. The naively selected sites are heavily biased against two patients, CRC02 and CRC15, and toward one patient, CRC11. Among the FALAFL-chosen sites, the biases are again reduced. Note that CRC02 and CRC15 are the two patient samples underrepresented due to comparatively worse coverage than others, and they happen to be two of the three patients with primary tumor sites in the right colon. While the naive approach of site selection ignores this underrepresentation when choosing the features and creates biases against right colon cancer patients, FALAFL corrects for this bias and ensures a balanced representation of sites from CRC patients of all three primary sites.

To further demonstrate the ability of FALAFL to perform balanced feature selection, we also benchmark FALAFL against the greedy pairwise selection using the data of the left colon CRC subcohort. We perform greedy pairwise selection, which maximizes the number of shared sites between each pair of patients in the subcohort, as detailed in Section 2.5. The right panel of Fig. 6a provides clear evidence that FALAFL balances the number of sites shared across different pairs of patients, whereas the greedy pairwise approach selects drastically different numbers of sites for each patient pair. Additionally, as shown in the left panel of Fig. 6a, FALAFL selects a much greater number of sites to represent the left colon CRC subcohort than the greedy pairwise approach does: FALAFL outputs ∼1.35M sites for the left colon CRC subcohort, whereas there are only ∼0.47M sites shared across all four patients selected by the greedy pairwise approach. This result shows that FALAFL is able to not only select molecular features to maximally represent the entire patient cohort but also balance the information shared for each pair of patients without substantially favoring any patient pairs.

### 3.4 FALAFL helps discover cohort-wise universal behavior in methylation changes

We use FALAFL to select CpG sites representative of the left colon CRC subcohort and the entire CRC cohort, respectively, as described in Section 2.1. We use the measures described in Section 2.2 and 2.3 to evaluate the universal lineage-informativeness of the FALAFL-chosen sites for both cohorts. The mean JS distance is the natural threshold to differentiate lineage-informative and uninformative sites in a balanced manner—the focus of FALAFL. Similarly, the mean perfect correlation is the natural threshold for assessing the universality of these sites’ lineage-informativeness. Universally lineage-informative sites determined by these natural thresholds substantially differ from the uninformative sites in their genomic placement, implying that these thresholds point to biological differences (see Fig. 7).

**Figure 4.**
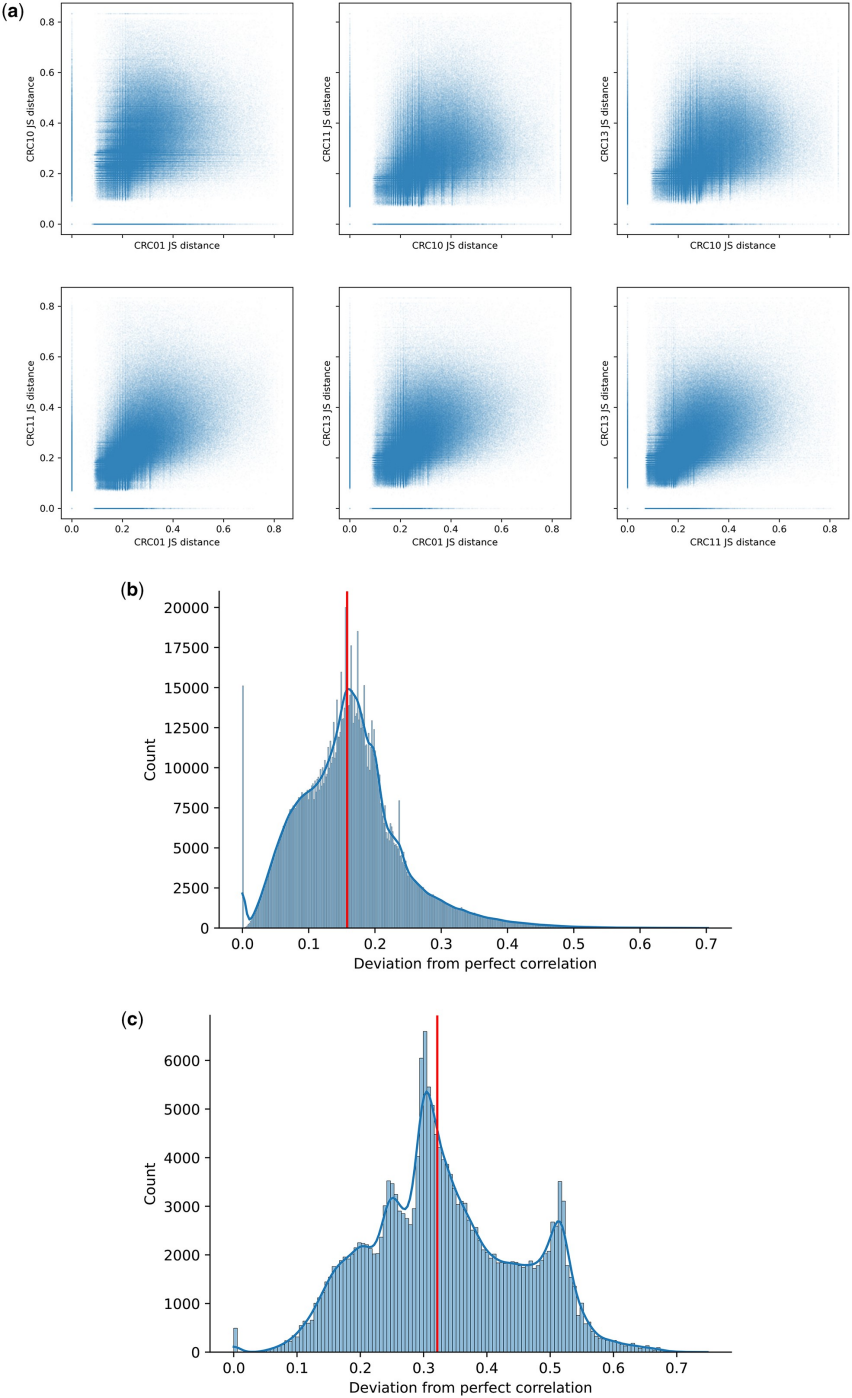
Correlation of lineage-informativeness of FALAFL-chosen CpG sites. (a) Pairwise scatter plot showing the lineage-informativeness correlation of FALAFL-selected sites for each patient pair of the left colon CRC subcohort. (b) Deviation from perfect correlation for four left colon CRC patients. The red vertical line indicates that the median deviation from perfect correlation is 0.1589. (c) Distance from the line of perfect correlation for all nine patients. The red vertical line indicates the median deviation from perfect correlation is 0.3216.

As depicted in Fig. 4a, the lineage-informativeness level of FALAFL-chosen CpG sites for the left colon CRC subcohort are highly correlated among all pairs of patients. As described in Section 2.3, we carry out further examination of the universality of lineage-informativeness levels for all patients in the left colon CRC subcohort. As shown in Fig. 4b, the majority of CpG sites display lineage-informativeness levels only slightly deviating from the line of perfect equality, with a median normalized shortest distance of 0.1589. We also evaluate the universality of lineage-informativeness of FALAFL-selected CpG sites for the entire CRC cohort of nine patients. Among the FALAFL-selected 195 809 sites, the median of the deviation from perfect correlation is 0.322, as demonstrated in Fig. 4c. This result shows that the behaviors of CpG sites are worse across a more diverse patient cohort, but nonetheless, there are still 11 916 out of the 195 809 FALAFL-selected CpG sites with deviations from perfect correlation smaller than 0.1589 (the median deviation for the left colon CRC subcohort). These results manifest the ability of FALAFL to discover universally lineage-informative CpG sites in both homogeneous patient cohorts and more diverse cohorts.

### 3.5 FALAFL better identifies sites that are universally lineage-informative

**Figure 5.**
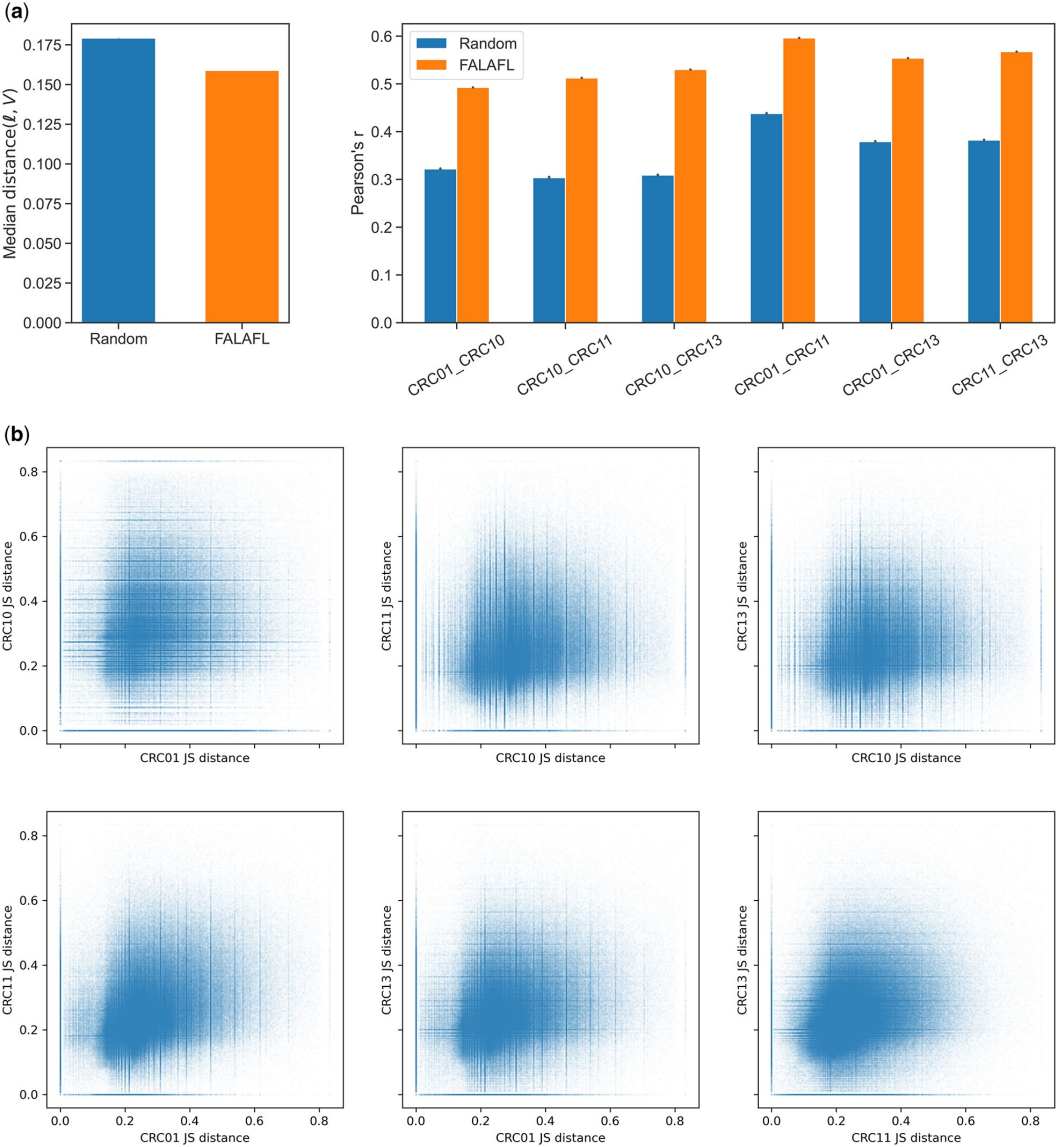
Benchmarking FALAFL against random CpG site selection. (a) Comparing random site selection and FALAFL. An equal number of sites in the FALAFL output are randomly selected from the unpreprocesed sitesof the left colon CRC subcohort. This is repeated five times. The left panel shows the median distance(ℓ,V) (i.e. deviation from perfect correlation) among the randomly selected sites versus that of the FALAFL-selected sites. The right panel shows the Pearson correlation of randomly selected sites versus the FALAFL-selected sites for each patient pair. Both panels demonstrate that FALAFL-selected sites better support the universality of lineage-informativeness than the randomly selected sites. (b) Correlation of lineage-informativeness of randomly chosen CpG sites across each pair of left colon cancer patients. See Fig. 4a for comparison.

We further show that the balanced site selection enabled by FALAFL substantially helps identify CpG sites that are universally lineage-informative. For this purpose, we first perform the random selection of CpG sites from the left colon CRC subcohort unpreprocessed sitesas described in Section 3.2. Specifically, we randomly choose 1 346 130 sites to match with the number of sites chosen by FALAFL for the left colon CRC subcohort, and we repeat the random sampling five times, as detailed in Section 2.5. As can be observed in Fig. 5b and the right panel of Fig. 5a, the Pearson correlation of the JS distance of randomly selected sites is consistently lower than that of FALAFL-selected sites for each patient pair. In the left panel, it is also observed that the median distance(ℓ,V) of the JS distance of randomly selected sites are consistently higher than that of FALAFL-selected sites.

**Figure 6.**
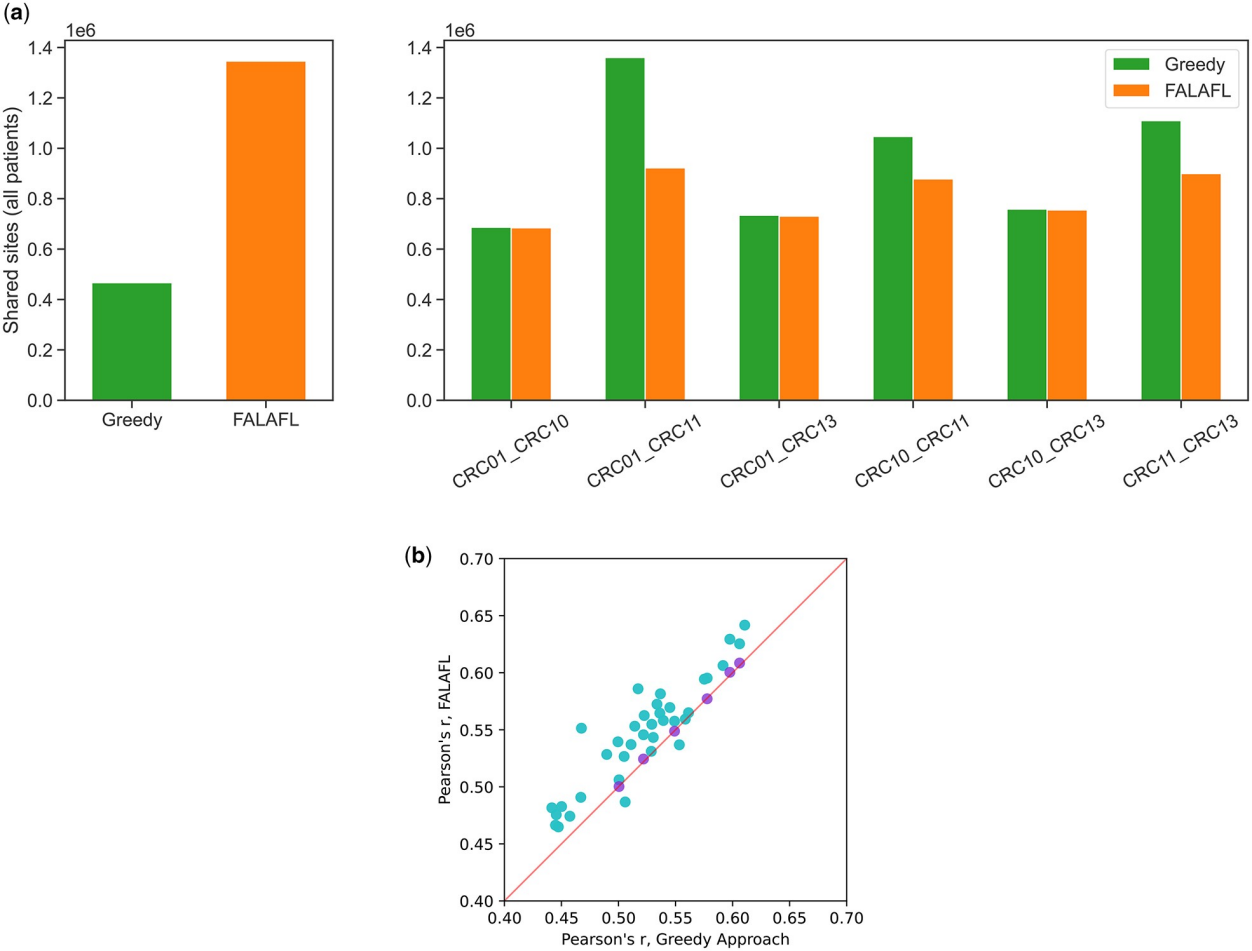
Benchmarking FALAFL against the greedy approach for CpG site selection. (a) Comparing the greedy approach and FALAFL for balanced feature selection. The left panel shows the number of CpG sites shared by all pairs of left colon CRC patients versus FALAFL-selected sites. The right panel shows the CpG sites present in at least 50% of cells in both patients for each patient pair, selected by the greedy approach versus that by FALAFL. (b) Comparing the greedy approach against FALAFL for correlation of lineage-informativeness for each patient pair. The *x*-axis is Pearson correlation of lineage-informativeness of greedy-selected CpG sites for each patient pair. The *y*-axis is Pearson correlation of lineage-informativeness of FALAFL-selected CpG sites for each patient pair. The *y*-coordinates of the purple points are Pearson correlation of lineage-informativeness of CpG sites selected by FALAFL in each pair of left colon CRC patients. The y-coordinates of the turquoise points are Pearson’s correlation of lineage-informativeness of CpG sites selected by FALAFL in the entire CRC cohort, again measured for each patient pair. The red line is the diagonal y=x.

We also compare the universality of lineage-informativeness for CpG sites selected using the pairwise greedy approach against FALAFL-selected CpG sites. Interestingly, FALAFL-selected CpG sites exhibit consistently higher correlation of lineage-informativeness (evaluated by Pearson correlation) across all patient pairs in the entire CRC cohort. As demonstrated in Fig. 6b, among the 36 pairs of nine patients, all but two patient pairs have increased Pearson correlations of lineage-informativeness for FALAFL-selected CpG sites for the entire CRC cohort of nine patients than sites selected using the greedy pairwise approach. The CpG sites selected by FALAFL for the left colon CRC cohort of four patients also demonstrate an increased level of correlation of lineage-informativeness, though the improvement is trivial. These benchmarking results demonstrate that the FALAFL-selected CpG sites consistently exhibit a higher level of universality for lineage-informativeness compared to sites chosen using the random approach or the greedy pairwise approach.

### 3.6 FALAFL-identified universally lineage-informative CpG sites reveal non-stochasticity of CpG methylation alterations in CRC progression

To understand the implications of universally lineage-informative and lineage-uninformative CpG sites, we stratify CpG sites by their universal lineage-informativeness in each patient pair; see Section 2.4. We identify universally lineage-informative and uninformative sites for the left colon CRC subcohort of four patients and the entire CRC cohort. Below, we characterize CpG sites with universal lineage-informativeness and uninformativeness to shed light on the commonality of methylation changes in colorectal cancer progression.

**Figure 7.**
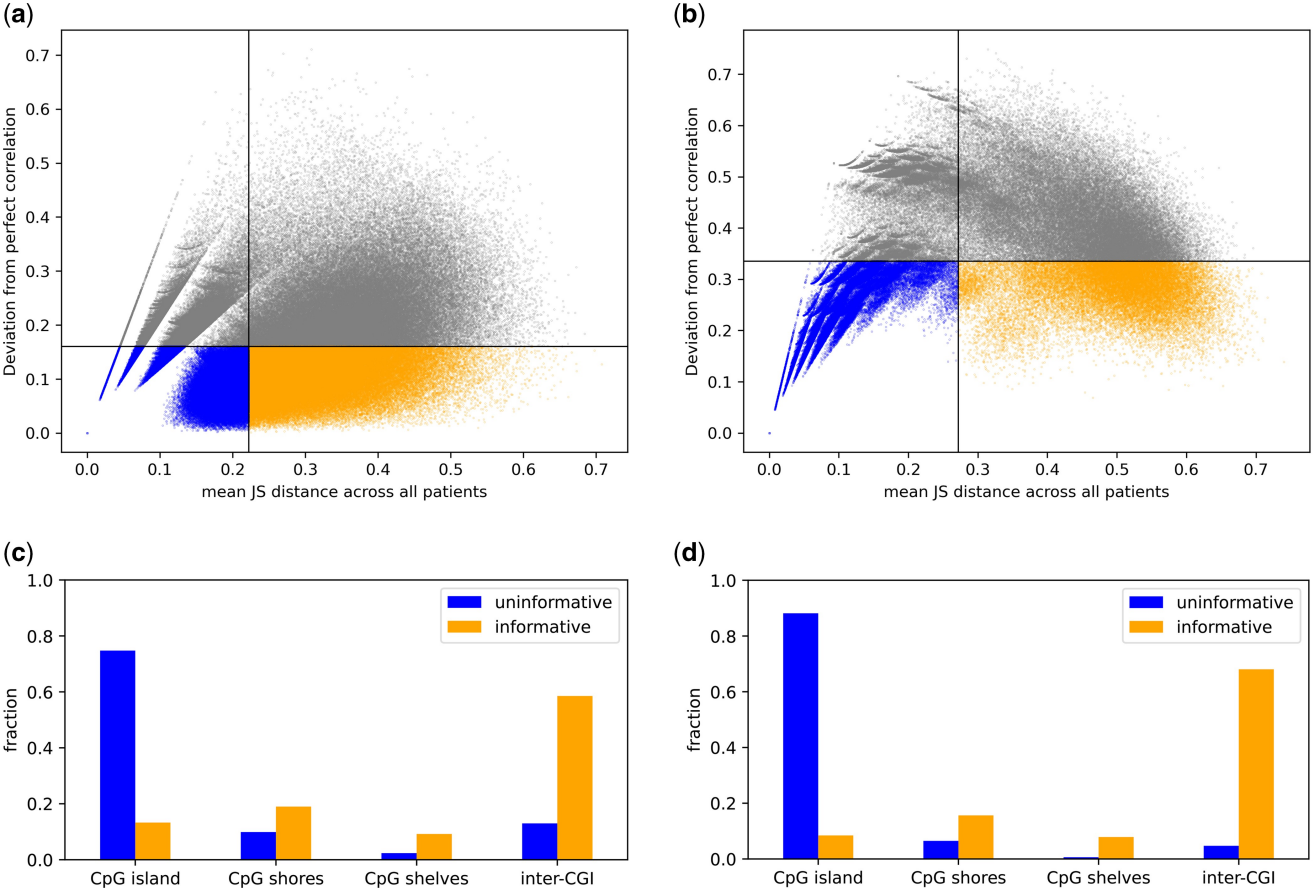
Characterization of FALAFL-identified universally lineage-informative and uninformative CpG sites in the left colon CRC subcohort and the entire CRC cohort. Universally lineage-informative sites are orange, while universally lineage-uninformative sites are blue. The remaining sites are gray. (a) Scatter plot showing mean JS distance against deviation from perfection correlation of each FALAFL-selected CpG site for the left colon CRC subcohort. The vertical line marks the mean of mean JS distance across all patients, which is 0.2224. The horizontal line marks the mean deviation from perfect correlation, 0.1671. (b) Scatter plot showing mean JS distance against deviation from perfection correlation of each FALAFL-selected CpG site for the entire CRC cohort. The vertical line marks the mean of mean JS distance across all patients, which is 0.2717. The horizontal line marks the mean deviation from perfect correlation, which is 0.3355. (c) Bar plot showing the fractions of universally lineage-informative and uninformative CpG sites in left colon CRC subcohort located in CpG island, shore, shelf, and inter-CGI regions. (d) Bar plot showing the fractions of universally lineage-informative and uninformative CpG sites in the entire CRC cohort located in CpG island, shore, shelf, and inter-CGI regions.

In the left colon CRC subcohort of four patients, FALAFL selects 1 346 130 sites. As shown in Fig. 7a, among all the selected sites, the mean of the mean JS distance across all patients is 0.2224, and the mean deviation from perfect correlation is 0.1671. Based on the definitions in Section 2.4, 321 006 sites are universally lineage-informative and 384 937 universally uninformative in the left colon CRC subcohort. In the CRC patient cohort of nine patients, FALAFL selects 195 809 sites to represent the entire cohort. As indicated in Fig. 7b, among the 195 809 sites, the mean value of mean JS distances across all patients is 0.2717, and the mean deviation from perfect correlation is 0.3355. In the entire CRC patient cohort, 31 378 sites are deemed universally lineage-informative and 76 189 universally uninformative.

Next, we categorize the universally lineage-informative and uninformative sites based on their genomic locations. Specifically, we map the sites to CpG islands [CGI, regions where CpG dinucleotides are over-represented (Cross and Bird 1995)], CpG shores [2 kb-long regions flanking both ends of CGIs (Irizarry *et al.* 2009)], CpG shelves [2 kb-long regions outside of CpG shores (Irizarry *et al.* 2009)], and inter-CGI regions. As demonstrated in Fig. 7c and d, in both the left colon CRC subcohort and the entire CRC cohort, universally lineage-informative sites are enriched in inter-CGI regions, whereas universally uninformative sites are predominantly localized in CpG islands. These results elucidate the non-stochastic nature of methylation changes in colorectal cancer progression and attest to the ability of FALAFL-selected molecular features to reveal interesting biological insights. The universally lineage-informative sites identified by FALAFL can may be further leveraged to gain insights into driver methylation events in cancer.

## 4 Conclusion

In this work, we present FALAFL (FAir muLti-sAmple Feature seLection), a novel fair molecular feature selection scheme aimed at ensuring algorithmic fairness in comparative patient profile analysis. Applied to the problem of fair selection of CpG sites within a cohort of colorectal cancer patients, our results demonstrate the ability of FALAFL to rapidly and robustly identify a maximal set of CpG sites well-represented across the entire patient cohort. Importantly, FALAFL ensures a balanced representation of molecular features across all patients while uncovering cohort-wise universal behaviors of methylation changes. Furthermore, our investigation into the FALAFL-selected sites reveals interesting enrichment of universally lineage-informative CpG sites in inter-CpG island regions, which indicates a degree of non-stochasticity of methylation changes in colorectal cancer progression. The application of FALAFL to a single-cell methylation sequencing dataset from a cohort of brain cancer patients exhibits the generalizability of these results. Our findings demonstrate the ability of FALAFL to identify lineage-informative sites and provide valuable insights into the underlying biological mechanisms of cancer progression. We aim to apply FALAFL to larger and more diverse patient cohorts and further explore its use in clinical settings.

## Supplementary Material

btaf237_Supplementary_Data

## Data Availability

The input data is available at https://github.com/algo-cancer/FALAFL.
