# Supplementary material for: Fair molecular feature selection unveils universally tumor lineage-informative methylation sites in colorectal cancer
Source: Bioinformatics. 2025 Jul 15;41(Suppl 1):i150–9. doi: 10.1093/bioinformatics/btaf237 (PMC12261425; doi:10.1093/bioinformatics/btaf237)
Supplement: btaf237_Supplementary_Data [file btaf237_supplementary_data.pdf]

Fair molecular feature selection unveils universally tumor lineage-informative methylation sites in colorectal cancer Supplementary Materials

Xuan Cindy Li[1,2,†], Yuelin Liu[1,4,†], Alejandro A. Schäffer[1], Stephen M. Mount[2,3], S. Cenk Sahinalp[1,*]

[1]Cancer Data Science Laboratory, Center for Cancer Research, National Cancer Institute, NIH, Bethesda, MD 20892, USA
[2]Program in Computational Biology, Bioinformatics, and Genomics, University of Maryland, College Park, MD 20742, USA
[3]Department of Cell Biology and Molecular Genetics, University of Maryland, College Park, MD 20742, USA
[4]Department of Computer Science, University of Maryland, College Park, MD 20742, USA
[*]Corresponding author. cenk.sahinalp@nih.gov
[†]Joint first authors.

S1 Additional information of metastatic CRC patients from Bian *et al.* data set

Patient	Primary site	# Cells
CRC01	Left Colon	160
CRC02	Right Colon	39
CRC04	Right Colon	65
CRC10	Left Colon	114
CRC11	Left Colon	228
CRC12	Rectum	22
CRC13	Left Colon	185
CRC14	Rectum	29
CRC15	Right Colon	50

Table S1: Data summary of the Bian *et al.* metastatic CRC cohort [1].

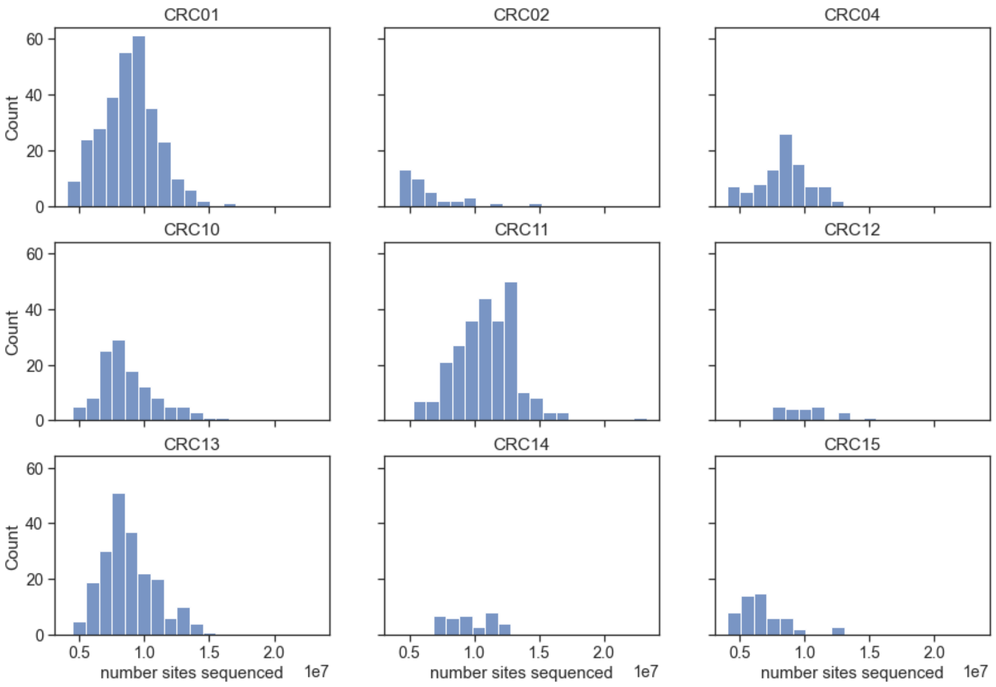

Figure S1: Distribution of CpG site coverage of the Bian *et al.* metastatic CRC cohort. The distribution plots show the number of CpG sites sequenced in each cell on the x-axis and the number of cells on the y-axis.

S2 Runtime and robustness of FALAFL

# Patients	Instance 1	Instance 2	Instance 3
10	8.14	8.43	8.59
20	152.01	138.79	149.79
30	413.25	583.07	500.15
40	667.42	637.15	761.31
50	5224.93	4517.22	5763.57

Table S2: Time (in seconds) spent to obtain the optimal solution using FALAFL on randomly generated input binary matrices. $q = 0.75$.

# Patients	Instance 1	Instance 2	Instance 3
10	17.22	18.54	11.17
20	53.09	85.86	75.72
30	305.08	129.50	301.81
40	382.56	407.65	346.48
50	505.20	255.23	256.83

Table S3: Time (in seconds) spent to obtain the optimal solution using FALAFL on perturbed real patient data. $q = 0.75$.

q	Instance 1	Instance 2	Instance 3
0.6	14.28	13.87	13.05
0.7	13.13	11.93	11.97
0.8	13.25	12.31	11.92
0.9	13.51	12.23	12.05

Table S4: Time (in seconds) spent to obtain the optimal solution using FALAFL on perturbed real patient data for different choices of q. Number of patients in the input is 10.

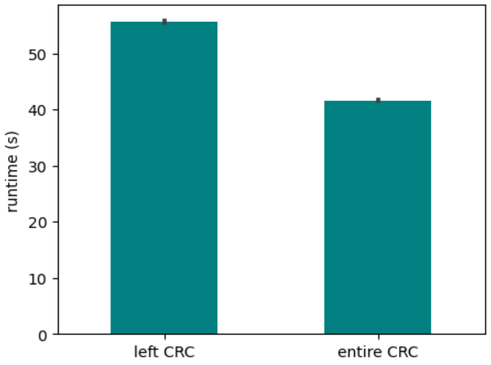

(a)

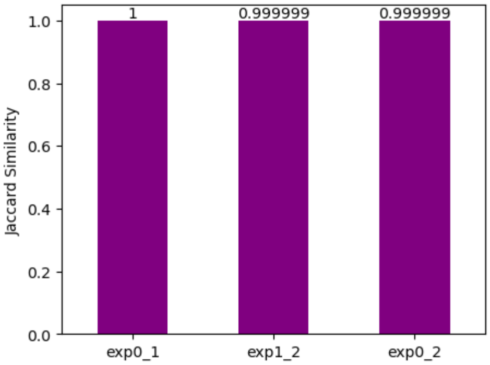

(b)

Figure S2: FALAFL runtime and robustness on real data. (a) Bar plot showing the mean FALAFL runtime of feature selection for the left colon CRC subcohort and the entire CRC cohort. The black error bars indicate the standard deviation of the runtime of all three instances. **(b)** Bar plot showing the Jaccard index of the FALAFL output for each pair of the shuffled inputs.

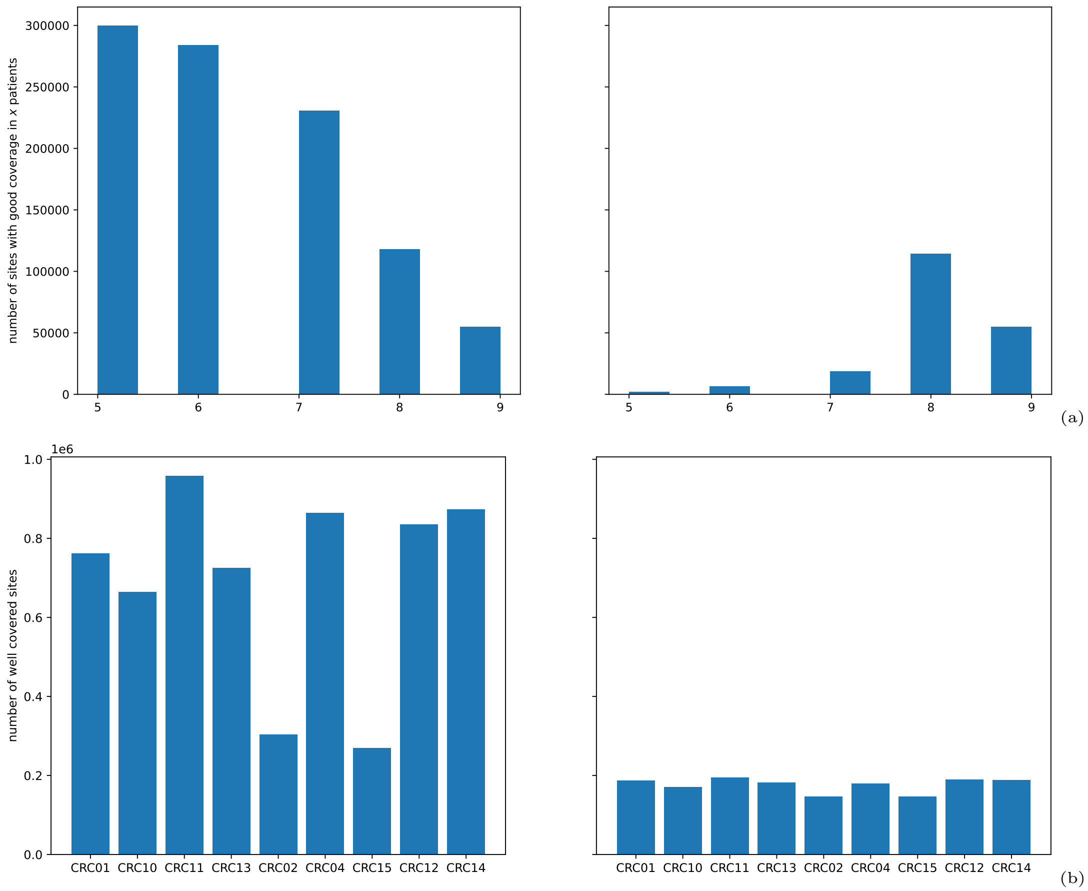

Figure S3: `FALAFL` reduces bias against or towards individual patients in the entire CRC cohort. **(a)** Bar plot showing the number of sites with good coverage in different number of patients. **(b)** Bar plot showing the number of sites with good coverage in each patient sample. The left panels show the results from naively selecting sites with good coverage in 4 or more patients. The right panels show the results from `FALAFL`-chosen sites.

S3 Applying `FALAFL` to glioma patient data

In addition to the context of colorectal cancer, we also explore applying `FALAFL` to the study of DNA methylation changes in glioma tumor evolution. We applied `FALAFL` to a single-cell reduced-representation bisulfite sequencing (scRRBS) data set [2], which consists of the two common types of glioma patients, IDH-mutant (IDH-MUT) and IDH-wildtype glioblastoma (GBM). Although this data set is also DNA methylation sequencing, it is obtained through a protocol different from the Bian *et al.* colorectal cancer data set.

S3.1 Data Processing

S3.1.1 Read Mapping and Methylation Calling

We performed demultiplex, alignment, and methylation calling with the pipeline provided by the original authors [2], with the following versions of the software involved `snakemake 6.8.2`, `bedtools 2.30.0`, `bowtie 2-2.4.4`, `bismark 0.23.0`, `samtools 1.13`. The original authors have used older versions of the software, namely `bismark 0.14.5`, `bedtools 2.25.0`, and `samtools 1.3.1`. The pipeline discards unmapped and ambiguous reads so that only uniquely mapped reads are considered for CpG methylation calling.

S3.1.2 Quality Control and Data Filtering

Given the methylation calls and read supports for the unique CpG sites, we perform some initial quality control and data cleaning. Specifically, we remove cells with coverage in fewer than 50,000 unique CpG sites and remove CpG sites on (i) unlocalized or unplaced fragments, (ii) sex chromosomes, (iii) mitochondrial DNA, and (iv) the Epstein-Barr virus (EBV) genome. We further remove CpG sites with coverage in only one cell in a single patient, as they will hold no phylogenetic signal. We also collapsed the cells from each group of technical replicates into one sample.

With the single-cell methylation calls obtained above for each of the 14 patients in the Chaligne *et al.* cohort, we perform data filtering. Specifically, for each patient, we remove cells with fewer than 200,000 unique CpG sites detected, and remove CpG sites (i) with coverage in fewer than 10% of the remaining cells, (ii) on unlocalized or unplaced fragments, (iii) on sex chromosomes, (iv) on mitochondrial DNA, or (v) within chromosome 21 centromeric region (chr21:9825000-9828000).

The number of cells and CpG sites post-filtering is shown in Table S5. We exclude MGH129, MGH211, and MGH201 from further analysis as they have fewer than 20 cells with sufficient coverage, leaving us 11 total patients. The distribution of the number of unique CpG sites covered per cell post-filtering are demonstrated in Figure S4.

Classification	Patient	Has Normal Control	Number of cells	Number of sites
GBM	MGH105	✓	198	275,365
	MGH115	✗	48	3,096,312
	MGH121	✗	133	573,291
	MGH122	✗	48	1,581,864
	MGH124	✗	42	716,959
	MGH129	✗	11	620,092
	MGH211	✗	14	1,735,183
IDH-MUT	MGH64	✗	60	1,280,600
	MGH45	✗	32	707,538
	MGH135	✗	60	598,163
	MGH107	✗	41	379,827
	MGH142	✗	174	587,716
	MGH201	✗	2	236,524
	MGH208	✗	256	1,098,332

Table S5: Input data dimensions. The data is obtained after first-pass filtering of (1) cells with coverage in fewer than 200,000 unique CpG sites and (2) sites present in fewer than 10% of the remaining cells across patients in the Chaligne *et al.* [2] glioma data set. We exclude MGH129, MGH211, and MGH201 from further analysis due to the low number of cells with sufficient read coverage.

GBM Patients:

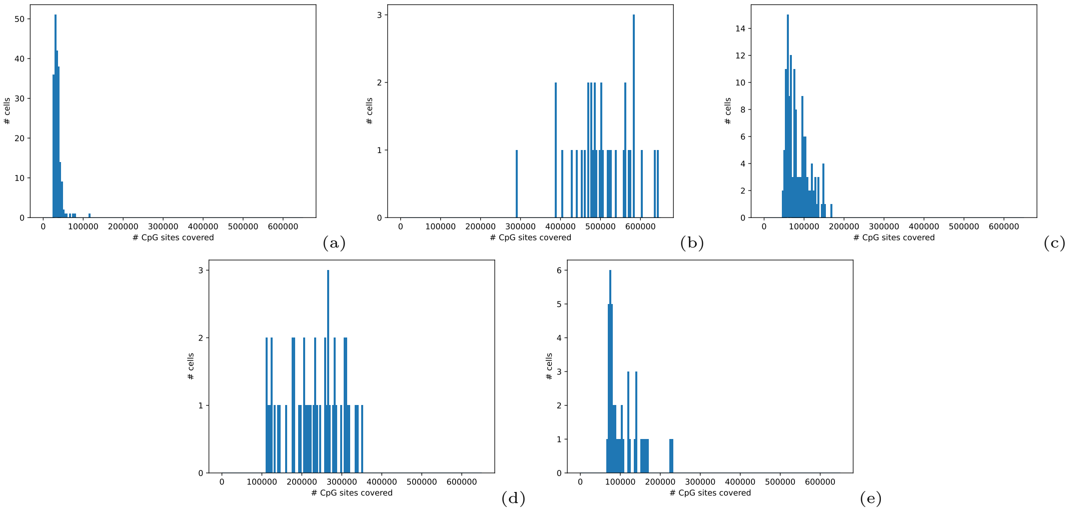

IDH-MUT Patients:

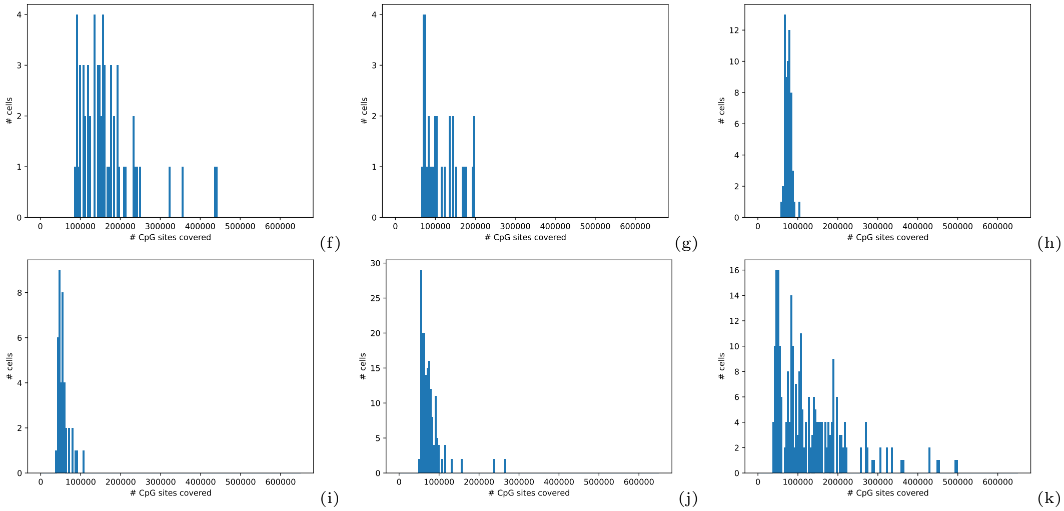

Figure S4: Distribution of the number of unique CpG sites covered per single cell. **(a)** MGH105, **(b)** MGH115, **(c)** MGH121, **(d)** MGH122, **(e)** MGH124, **(f)** MGH64, **(g)** MGH45, **(h)** MGH135, **(i)** MGH107, **(j)** MGH142, and **(k)** MGH208.

S3.2 Applying FALAFL on Chaligne *et al.* [2] glioma data set

We established three patient cohorts based on the tumor types: all patients, IDH-MUT, and GBM (IDH-wildtype). For all 11 patients passing data filtering as described in Section S3.1.2, the number of all sites combined is 4,784,835. For the subsequent preprocessing step, we choose $p = 0.1$ and $k = 5$, where the choice of k is the floor of half of the cohort size. Also note that compared to the the Bian *et al.* [1] data set, the Chaligne *et al.* [2] glioma data set is significantly sparser, as can be observed in Figure S4. As a result, we empirically choose a smaller value for p to avoid trimming away the vast majority of information in the preprocessing step. The dimension of the resulting matrix (i.e., $\overline{S}$ as described in Section 2.1) after the preprocessing steps is $11 \times 511,021$. For the IDH-MUT cohort, the number of all sites combined is 3,267,783, and we choose $p = 0.1$ and $k = 3$

for the preprocessing parameters. The dimension of the resulting matrix is $6 \times 327,060$. For the GBM cohort, the number of all sites combined is 3,446,993, and we choose $p = 0.1$ and $k = 2$ for the preprocessing parameters. The dimension of the resulting matrix is $5 \times 1,770,911$. For all three cohorts (all patients, IDH-MUT, and GBM), we chose parameter $q = 0.75$, and obtained 1,517, 20,572, and 280,449 sites in the `FALAFL` output after 29.92, 12.45, and 57.66 seconds, respectively.

S3.3 Analyzing `FALAFL`-chosen sites

We analyzed the `FALAFL`-chosen sites based on tumor lineage informativeness with the following analyses similar to those described in Section 2 of this paper.

S3.3.1 Generating tumor lineage trees and computing tumor lineage informativeness

We apply `Sgootr`[3] to the filtered data from 11 patients of the Chaligne glioma cohort with the following parameters to obtain the tumor lineage trees:

`DEFAULT_CNA=2, E=.01, SITE_COVERAGE_THRESHOLD=.001, CELL_COVERAGE_THRESHOLD=.001, STATUS_CONFIDENCE_THRESHOLD=1, P00=.33, P10=.33, P11=.33, P=.5, D0010=.5, D0011=1, D1011=.5, ALGO=N, PARTITION_VALIDITY_THRESHOLD=.1, MINIMUM_SUBTREE_SIZE=.05`.

After obtaining the tumor lineage trees, we computed the JS-distance-based lineage-informative sites based on the tree structure as described in Section 2.2 of this paper and as described in [3].

S3.3.2 Benchmarking against naive feature selection approaches

We benchmark `FALAFL` results obtained from the GBM (IDH-wildtype) subcohort, which is the subcohort with the best overall coverage, against the three naive feature selection approaches described in Section 2.5. Specifically, we conducted the following: (1) we selected sites that are well covered in at least 2 of the 5 patients in the GBM subcohort; (2) we performed greedy pairwise selection to select sites that are well covered for each pair of patients; (3) we randomly picked 280,449 sites, the same number of sites `FALAFL` output for the GBM subchort, from all 4,784,835 sites sequenced in the GBM subcohort.

S3.4 Interpreting `FALAFL`-chosen sites for glioma cohort and subcohorts

S3.4.1 `FALAFL` reveals cohort-specific behaviors in the lineage informativeness of methylation changes in glioma

As described in Section S3.2, while the number of sites identified by `FALAFL` was reasonably high for the IDH-mutant (IDH-MUT) subcohort (20,572 `FALAFL`-chosen sites) and was even higher for the IDH-wildtype (GBM) subcohort (280,449 `FALAFL`-chosen sites), when we combined the two subcohorts and applied `FALAFL` , we ended up with a small number of sites (1,517 `FALAFL`-chosen sites).

We obtained the pairwise Pearson correlation of lineage-informativeness of the `FALAFL`-chosen sites in each of the three cohorts. The pairwise Pearson correlation for each patient pair is shown in Figure S7. We have two main observations: (1) the pairwise Pearson correlation of lineage informativeness is similar for sites chosen within the subcohorts and those obtained on the entire cohort despite the vast differences in number of sites, as seen in Figure S6; (2) calculated from the `FALAFL`-chosen sites for the entire glioma cohort, the GBM patient pairs demonstrate a higher level of correlation of lineage-informativeness than the IDH-MUT patient pairs, which has a level of correlation similar to that of between-subcohort pairs, as can be observed in Figure S6.

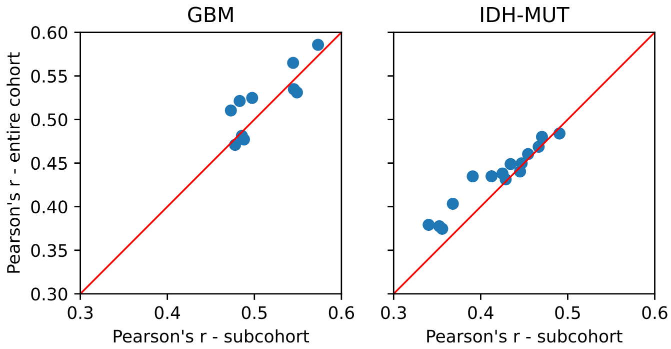

Figure S5: Pairwise Pearson correlation of lineage-informativeness calculated based on FALAFL-chosen sites for subcohort vs. the entire glioma cohort. Left panel: GBM subcohort vs. entire cohort. Right panel: IDH-MUT subcohort vs. entire cohort. Every dot represents a patient pair.

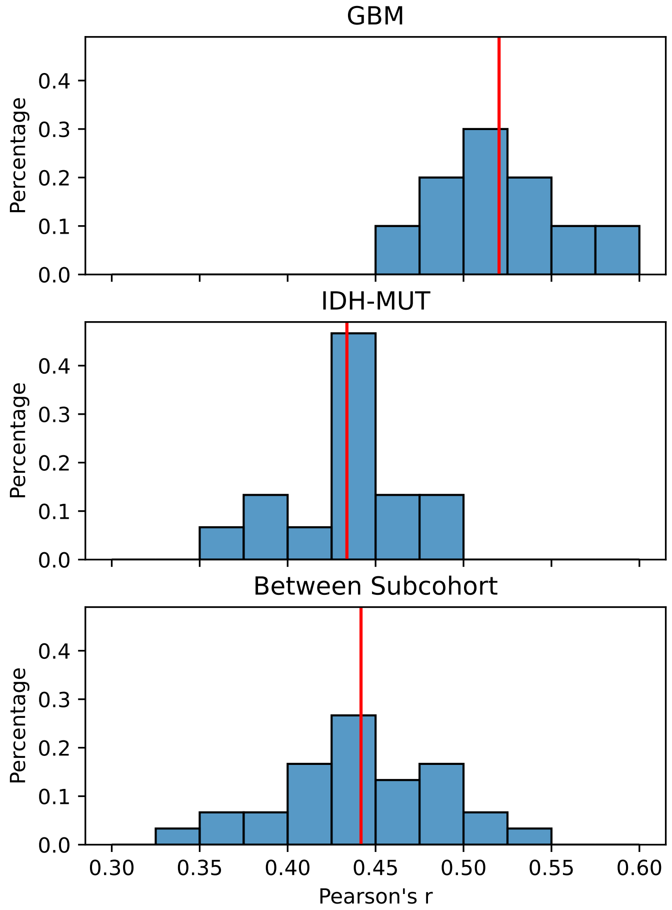

Figure S6: Pairwise Pearson correlation of lineage-informativeness calculated based on FALAFL-chosen sites for the entire glioma cohort. The panels from top to bottom show the pairwise correlation of GBM patient pairs, IDH-MUT pairs, and betwee-subcohort pairs. The red vertical line indicates the mean Pearson correlation for each type of patient pair. The mean Pearson correlation of GBM pairs is 0.5202, of IDH-MUT pair is 0.4337, and of between-subcohort pairs is 0.4446.

GBM Patients:

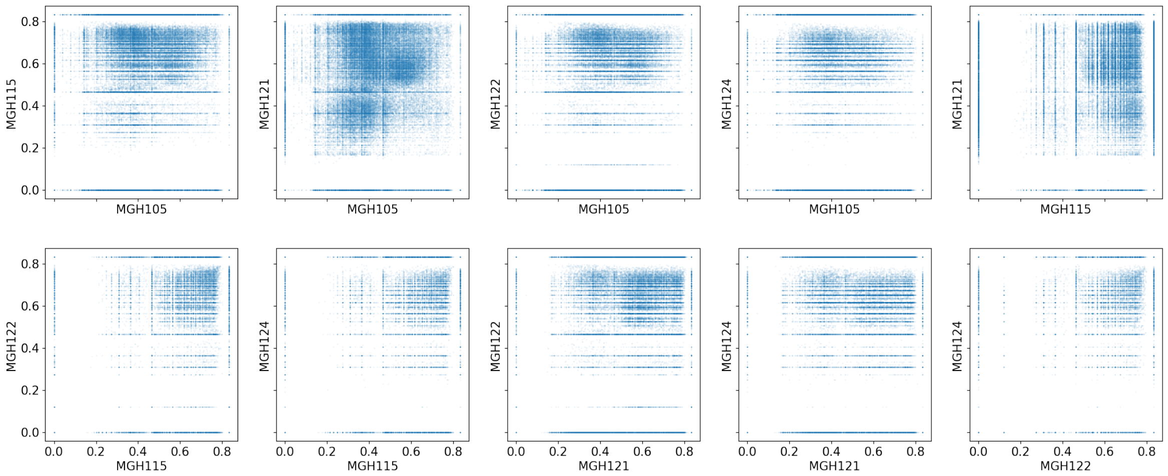

(a)

IDH-MUT Patients:

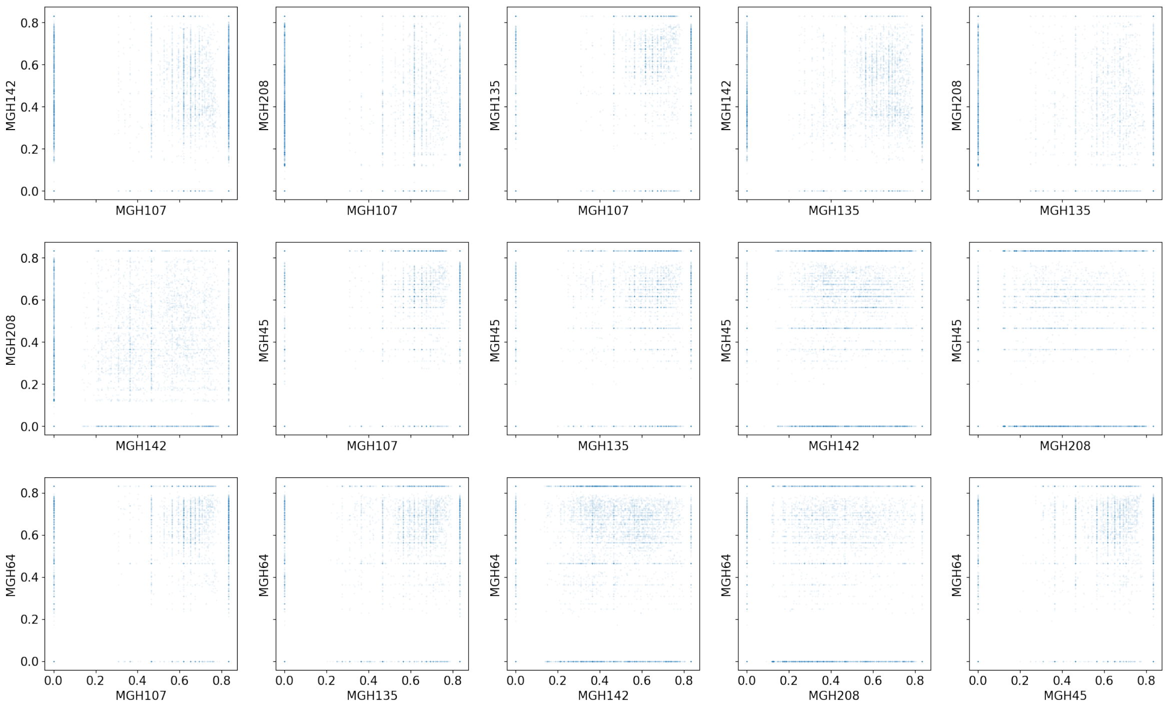

(b)

All patients:

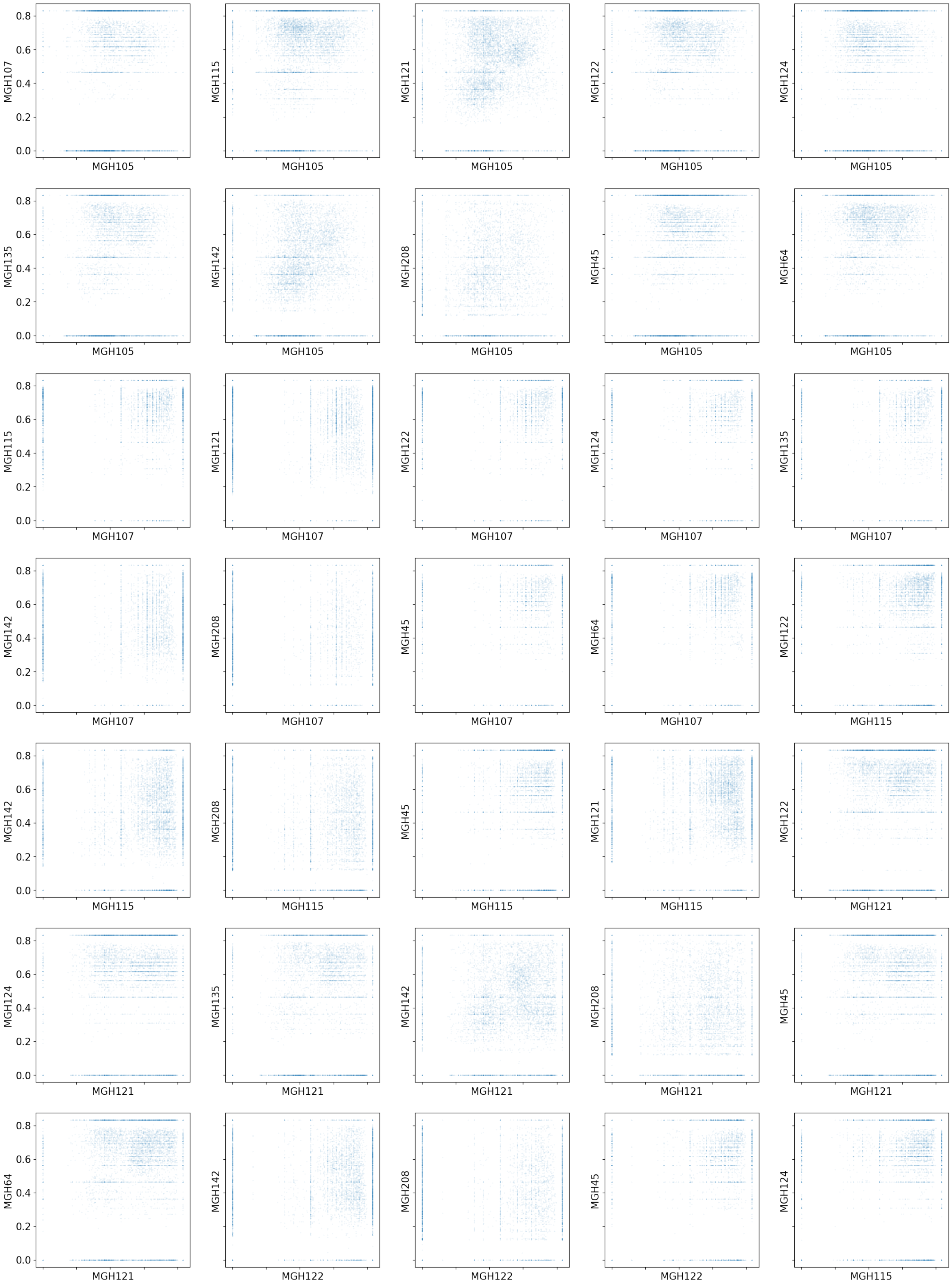

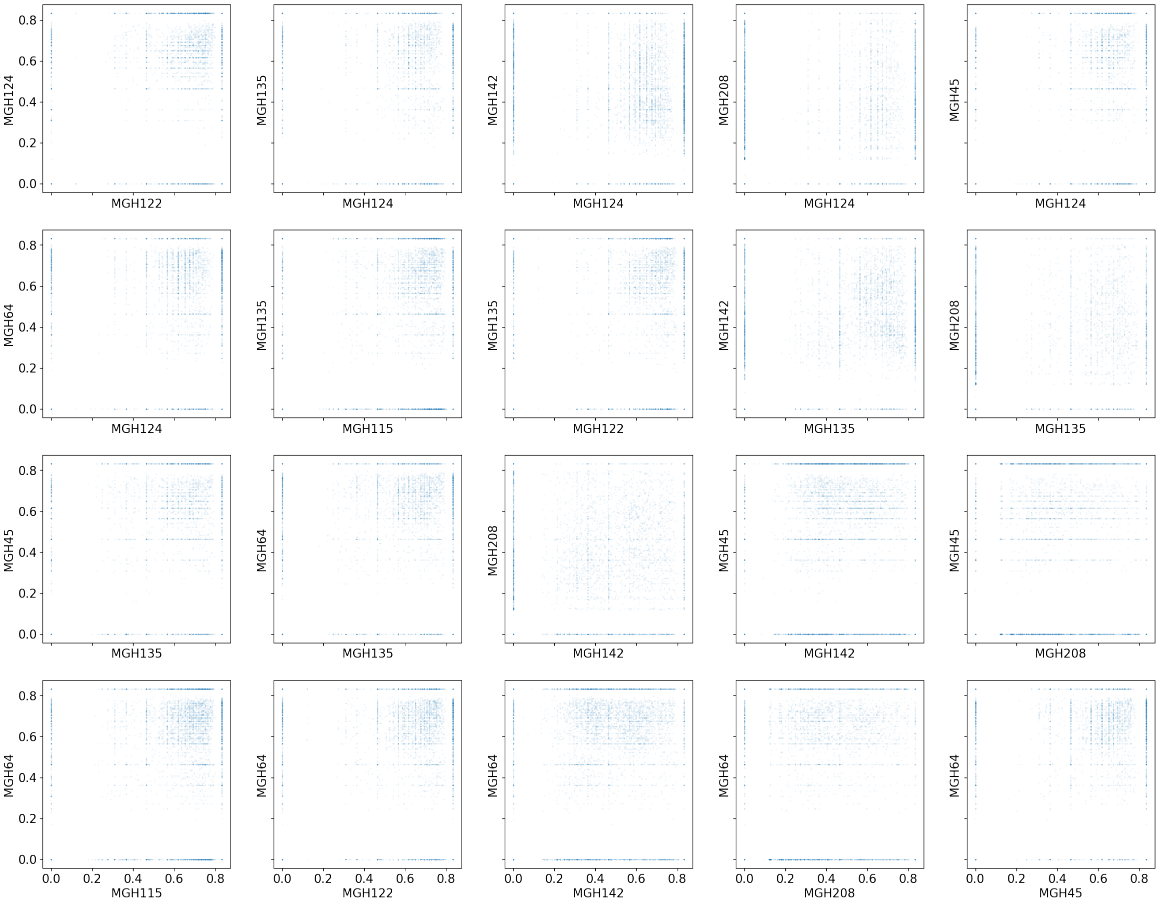

(c)

Figure S7: Pairwise pearson correlation for `FALAFL`-chosen sites for different cohorts. (a) GBM, **(b)** IDH-MUT, **(c)** All.

S3.4.2 `FALAFL` better identifies universally lineage-informative sites than naive approaches

Benchmarking against selecting sites covered in large fractions of patients. As can be seen in Figure S8(b), in the GBM (IDH-wildtype) subcohort, the naively chosen sites are heavily biased towards patient MGH115 and MGH122, both of which have notably more sites with good coverage than the other patients, whereas among `FALAFL`-chosen sites, the bias is reduced.

Benchmarking against greedy pairwise selection. The right panel of Figure S3.4.2 provides clear evidence that `FALAFL` balances the number of sites shared across different pairs of patients, whereas the greedy pairwise approach selects drastically different numbers of sites for each patient pair. Additionally, as shown in the left panel of Figure S3.4.2, `FALAFL` selects a much greater number of sites to represent the GBM subcohort than the greedy pairwise approach does: `FALAFL` outputs $\sim 280k$ sites for the GBM subcohort, whereas there are only $\sim 71k$ sites shared across all 5 patients selected by the greedy pairwise approach. Similar to our findings for the Bian *et al.* data set [1] in the main text, this result shows that `FALAFL` is able to not only select molecular features to maximally represent the entire patient cohort but also balance the information shared for each pair of patients without substantially favoring any patient pairs.

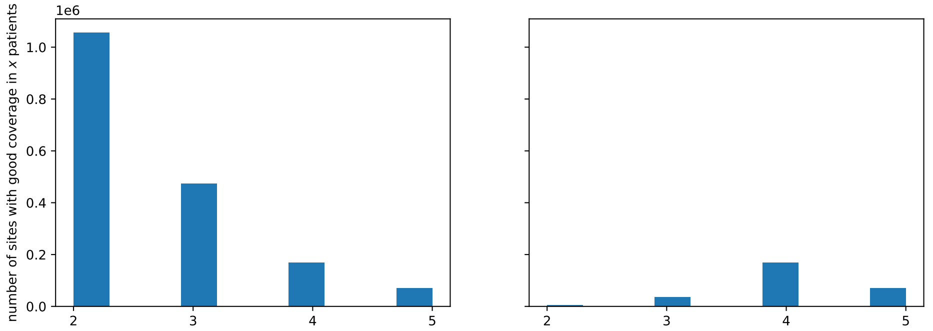

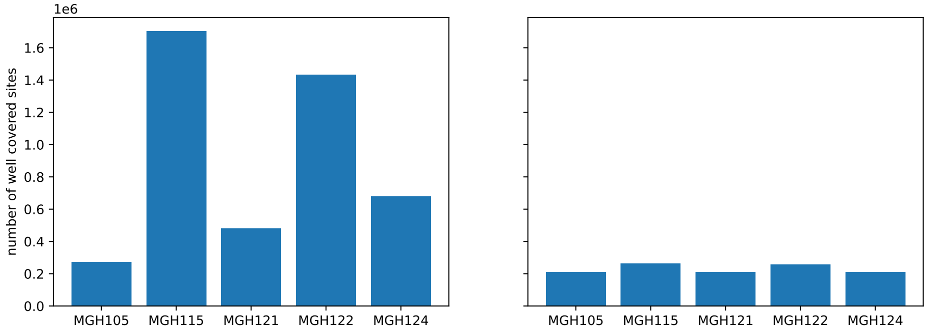

Figure S8: FALAFL reduces bias towards individual patients in the GBM (IDH-wildtype) subcohort. (a) Bar plot showing the number of sites with good coverage in different number of patients. **(b)** Bar plot showing the number of sites with good coverage in each patient sample. The left panels show the results from naively selecting sites with good coverage in 2 or more patients. The right panel shows the results from FALAFL-chosen sites.

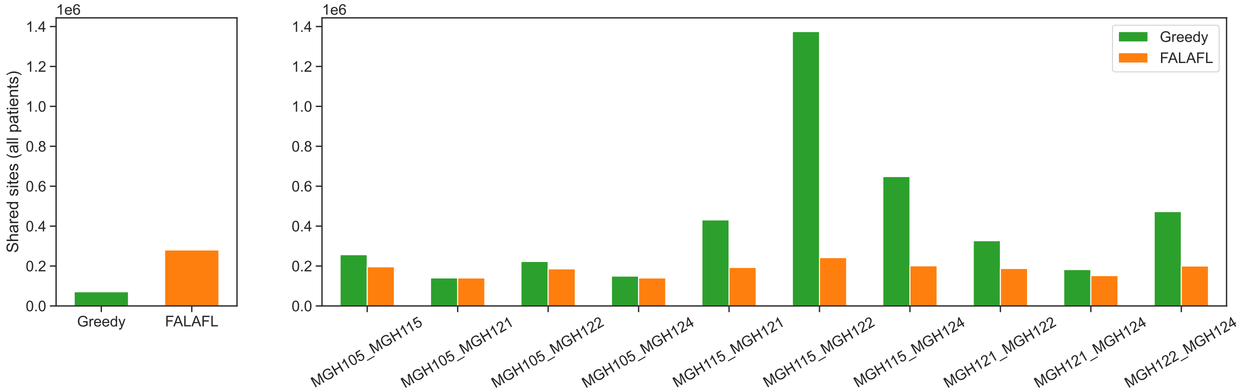

Figure S9: Benchmarking FALAFL against the greedy approach for CpG site selection in the GBM subcohort. Comparing the greedy approach and FALAFL for balanced feature selection. The left panel shows the number of CpG sites shared by all pairs of patients of the GBM subcohort selected by the greedy pairwise approach (effectively selecting sites shared by all patients) vs. FALAFL-selected sites. The right panel shows the CpG sites present in at least 10% of cells in both patients for each patient pair, selected by the greedy approach vs that by FALAFL.

Benchmaarking against random selection. As can be observed in Figure S3.4.2, the Pearson correlation of the JS distance of randomly selected sites is consistently lower than that of `FALAFL`-selected sites for each patient pair.

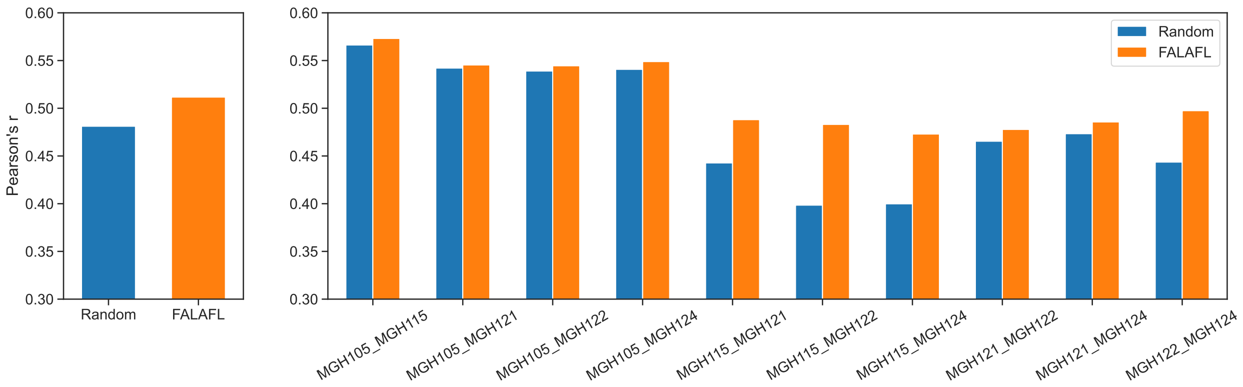

(a)

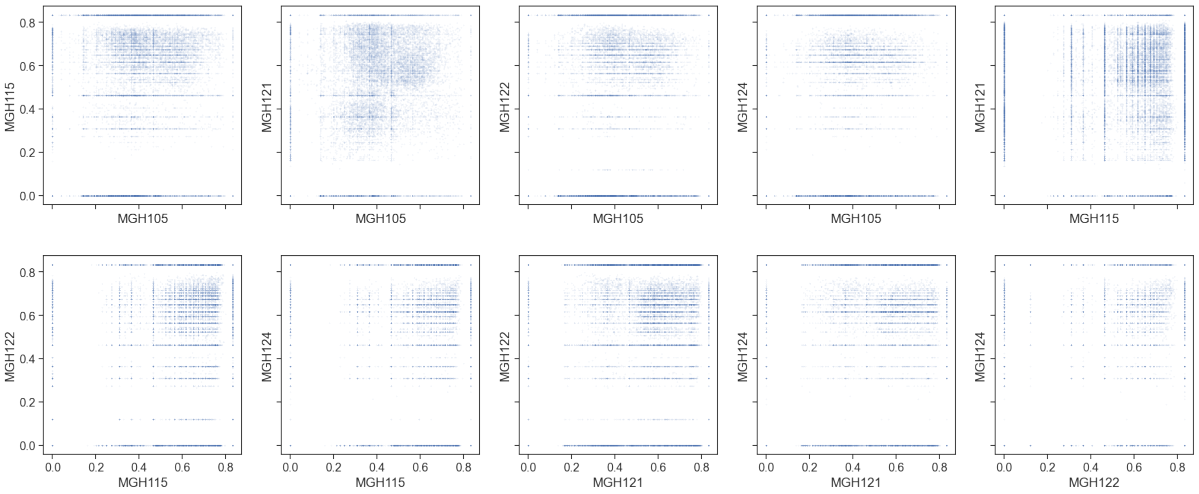

(b)

Figure S10: Benchmarking `FALAFL` against random CpG site selection. (a) Comparing random site selection and `FALAFL`. An equal number of sites in the `FALAFL` output are randomly selected from the `FALAFL` input matrix of the GBM subcohort. The left panel shows the mean pairwise Pearson correlation of randomly selected sites vs. that of the `FALAFL`-selected sites. The right panel shows the Pearson correlation of randomly selected sites vs. the `FALAFL`-selected sites for each patient pair. Both panels demonstrate that `FALAFL` selected sites better support the universality of lineage informativeness than the randomly selected sites. **(b)** Correlation of lineage-informativeness of randomly chosen CpG sites across each pair of left colon cancer patients. See Figure S7(a) for comparison.

References

[1] Shuhui Bian, Yu Hou, Xin Zhou, Xianlong Li, Jun Yong, Yicheng Wang, Wendong Wang, Jia Yan, Boqiang Hu, Hongshan Guo, et al. Single-cell multiomics sequencing and analyses of human colorectal cancer. *Science*, 362:1060–1063, 2018.

[2] Ronan Chaligne, Federico Gaiti, Dana Silverbush, Joshua S. Schiffman, Hannah R. Weisman, Lloyd Kluegel, Simon Gritsch, Sunil D. Deochand, L. Nicolas Gonzalez Castro, Alyssa R. Richman, Johanna Klughammer, Tommaso Biancalani, Christoph Muus, Caroline Sheridan, Alicia Alonso, Franco Izzo, Jane Park, Orit Rozenblatt-Rosen, Aviv Regev, Mario L. Suvà, and Dan A. Landau. Epigenetic encoding, heritability and plasticity of glioma transcriptional cell states. *Nature Genetics*, 53(10):1469–1479, 2021.

[3] Yuelin Liu, Xuan Cindy Li, Farid Rashidi Mehrabadi, Alejandro A Schäffer, Drew Pratt, David R Crawford, Salem Malikić, Erin K Molloy, Vishaka Gopalan, Stephen M Mount, et al. Single-cell methylation sequencing data reveal succinct metastatic migration histories and tumor progression models. *Genome Research*, 33:1089–1100, 2023.
